# Comparative effects of combined aerobic exercise-based interventions on metabolic, cardiovascular, and inflammatory markers in adult men: a systematic review and meta-analysis

**DOI:** 10.3389/fphys.2026.1810357

**Published:** 2026-04-16

**Authors:** Kai Gao, Yuncong Zhou, Chengyu Zhou, Lin Luo, Diandong Lang, Jutao Feng

**Affiliations:** 1 Strength And Conditioning Training College, Beijing Sports University, Beijing, China; 2 College of Sports and Health, Linyi University, Linyi, China; 3 Department of Sports Science, The University of Suwon, Hwaseong-si, Republic of Korea; 4 Department of Physical Education and Research, China University of Mining and Technology, Beijing, China; 5 Department of Physical Education, North China Electric Power University, Beijing, China; 6 Department of Martial Arts, Hebei Sports University, Hebei, China

**Keywords:** adult men, aerobic exercise, cardiovascular markers, inflammatory markers, meta-analysis

## Abstract

**Objective:**

The objective of this meta-analysis was to systematically assess and quantitatively synthesize the effects of aerobic exercise–based combined interventions on metabolic, cardiovascular, and inflammation-related outcomes in adult men.

**Methods:**

For this systematic review and meta-analysis of randomized controlled trials, systematic searches were conducted in PubMed, Embase, Web of Science, and the Cochrane Library from inception to December 20, 2025. Eligible studies compared aerobic exercise–based combined interventions (aerobic exercise delivered concurrently with at least one additional component, such as resistance training, nutritional supplementation, or adjunctive therapies) with control conditions. A random-effects model was applied for quantitative synthesis. Risk of bias was assessed using the Risk of Bias 2.0 tool. All statistical analyses were performed using Stata 15.

**Results:**

Fourteen randomized controlled trials involving 369 adult men were included. Pooled analyses suggested that aerobic exercise–based combined interventions were associated with reductions in BMI (WMD = −1.53, 95% CI −2.38 to −0.67) and changes in lipid parameters, including higher HDL (WMD = 2.37, 95% CI 0.72 to 4.02) and lower LDL (WMD = −9.25, 95% CI −15.16 to −3.34) and TC (WMD = −19.15, 95% CI −36.46 to −1.85). However, substantial between-study heterogeneity was observed across most outcomes (I² ranging from 76% to 99%). No statistically significant pooled effects were found for TG, SBP, DBP, or IL-6. Subgroup analyses showed variability in effect estimates across intervention types and populations, although several subgroup findings were based on single studies and should be interpreted cautiously.

**Conclusions:**

Aerobic exercise–based combined interventions may be associated with favorable changes in BMI and selected lipid parameters in adult men. However, the evidence is limited by substantial heterogeneity, small sample sizes, and reliance on combined intervention designs, which precludes clear attribution of effects to specific components. Further well-designed trials are needed to clarify the consistency and clinical relevance of these findings.

**Systematic Review Registration:**

https://www.crd.york.ac.uk/PROSPERO/view/CRD420261299383, identifier CRD420261299383.

## 1 Introduction

Globally, the prevalence of metabolic disorders and cardiovascular diseases continues to rise, posing significant public health challenges. Recent global burden estimates and updated guideline perspectives (2024–2026) further emphasize the growing impact of obesity, hypertension, and dyslipidemia as leading contributors to premature mortality and long-term healthcare burden (Yu et al., 2024; Moon et al., 2025). Adult males are particularly susceptible to metabolic and cardiovascular risk factors such as overweight, elevated blood pressure, and dyslipidemia due to lifestyle patterns, occupational stress, and behavioral factors (Chen et al., 2023; Zionskowski et al., 2025). These conditions frequently cluster and interact, thereby amplifying the risk of cardiovascular and cerebrovascular events (Quach et al., 2022). Consequently, identifying effective and sustainable non-pharmacological strategies remains a priority for this population (Husøy and Steiner, 2025).

Aerobic exercise, as a non-pharmacological intervention, is widely recommended for preventing and managing cardiometabolic disorders. Previous studies (Kaufmann et al., 2022; Asimakidis et al., 2025) suggest that aerobic exercise may reduce body mass index (BMI) and improve body composition through mechanisms such as increased energy expenditure, enhanced insulin sensitivity, and regulation of lipid metabolism. It may also exert beneficial effects on blood pressure and vascular function by improving vascular compliance and modulating autonomic balance (Cahuê et al., 2023; Selmi et al., 2023). In addition, aerobic exercise has been associated with changes in lipid profiles, including reductions in low-density lipoprotein (LDL), total cholesterol (TC), and triglycerides (TG), along with modest increases in high-density lipoprotein (HDL) (Hernández-García et al., 2024). However, recent evidence indicates that these effects are not always consistent across studies, and substantial heterogeneity has been reported, potentially reflecting differences in intervention design, population characteristics, and co-interventions (Ruscica et al., 2021). Interleukin-6 (IL-6), a key inflammatory marker linked to insulin resistance and endothelial dysfunction, may also respond to exercise; however, findings remain inconclusive.

In recent years, aerobic exercise–based combined interventions have received increasing attention. However, the current evidence base presents several important methodological and conceptual limitations. First, many existing systematic reviews and meta-analyses have pooled pure aerobic exercise and combined interventions within the same analytical framework (Xiao et al., 2020), making it difficult to distinguish the effects attributable to different intervention components. This limits interpretability, particularly given that multi-component interventions are increasingly used in real-world clinical and community settings (Laurindo et al., 2025). Second, most prior meta-analyses have included mixed-sex populations or disease-specific cohorts without conducting sex-stratified analyses. Given known sex differences in fat distribution, hormonal regulation, and inflammatory responses to exercise (Farhat et al., 2025; Fernandez-Escabias et al., 2025). the applicability of pooled estimates to adult male populations remains uncertain. Third, previous studies have often focused on isolated outcomes such as BMI or blood pressure, while integrated evaluation of metabolic, lipid, and inflammatory markers—now recognized as interconnected components of cardiometabolic risk—remains limited.

In the present study, we specifically focused on aerobic exercise–based combined interventions rather than pure aerobic exercise. This decision was made to avoid conflating single-modality and multi-component interventions, which may differ in both magnitude and mechanisms of effect. Given that contemporary exercise programs are often delivered in combination with resistance training, nutritional strategies, or adjunctive therapies, evaluating combined interventions as a distinct category may provide more clinically relevant insights.

Additionally, this study restricted inclusion to adult male participants. This approach was intended to reduce biological heterogeneity and minimize confounding related to sex-specific physiological differences in cardiometabolic and inflammatory responses to exercise. While this may limit generalizability, it allows for a more focused and internally consistent assessment of intervention effects within a relatively homogeneous population.

Given these considerations, a systematic synthesis focusing specifically on adult males and integrating anthropometric, hemodynamic, lipid, and inflammatory outcomes is warranted. To our knowledge, this study provides a focused evaluation of aerobic exercise–based combined interventions in adult men. The present meta-analysis aims to comprehensively assess their associations with BMI, SBP, DBP, HDL, LDL, TC, TG, and IL-6, with the goal of providing more nuanced and population-specific evidence for cardiometabolic risk management.

## 2 Materials and methods

This study was designed and reported in strict adherence to the Preferred Reporting Items for Systematic Reviews and Meta-Analyses (PRISMA) guidelines (Page et al., 2021), The protocol was registered prospectively, prior to the start of the review and meta-analysis, ensuring transparency and adherence to predefined methods, with the registration number: CRD420261299383.

### 2.1 Literature search strategy

Systematic searches were conducted in PubMed, Embase, Web of Science, and the Cochrane Library from database inception to December 20, 2025. To enhance comprehensiveness and reduce potential publication bias, additional searches were performed in gray literature sources and clinical trial registries, including ClinicalTrials.gov and the WHO International Clinical Trials Registry Platform (ICTRP). No language restrictions were applied. Searches combined Medical Subject Headings (MeSH) terms and free-text keywords, including “aerobic exercise,” “combined aerobic exercise,” “physical activity,” and “adult men,” with Boolean operators (“AND” “OR”) used to construct the search strategy. The terms were adapted according to each database’s indexing and syntax rules. A representative PubMed search was conducted as follows: (“Aerobic Exercise”[Mesh] OR “aerobic exercise”[tiab] OR “cardiorespiratory exercise”[tiab] OR “endurance training”[tiab]) AND (“Physical Activity”[Mesh] OR “physical activity”[tiab] OR “exercise”[tiab]) AND ((“Resistance Training”[Mesh] OR “resistance training”[tiab] OR “strength training”[tiab] OR “weight training”[tiab]) OR (“Dietary Supplements”[Mesh] OR “nutritional supplement*”[tiab] OR “dietary intervention*”[tiab] OR “nutrition”[tiab])) AND (“Adult”[Mesh] OR “adult men”[tiab] OR “male”[tiab] OR “men”[tiab]), limited to randomized controlled trials. Full search strategies for all databases are presented in **Supplementary Table 1**.

### 2.2 Inclusion and exclusion criteria

#### 2.2.1 Inclusion criteria

Study subjects were adult males (≥18 years old).

Interventions were required to be aerobic exercise–centered combined interventions, defined as programs in which aerobic exercise constituted the primary component (the dominant mode in frequency, duration, or intensity), delivered concurrently with at least one additional component (resistance training, nutritional supplementation, or adjunctive therapies such as electrical stimulation).

Control groups were categorized as either (1) non-exercise or usual care controls, or (2) aerobic exercise alone. These comparator types were analyzed separately where appropriate to avoid conflating distinct intervention contrasts.

Reporting at least one pre-specified outcome, including BMI, SBP, DBP, HDL, LDL, TC, TG, or IL-6.

Study design was randomized controlled trials.

#### 2.2.2 Exclusion criteria

Non-original research (reviews, conference abstracts, case reports).

Studies from which valid data cannot be extracted.

Interventions that are unclear or do not meet the definition of combined aerobic exercise.

Studies involving non-adult male populations or where gender data cannot be separated.

### 2.3 Data extraction

Two researchers independently screened the literature, first conducting an initial screening based on titles and abstracts, followed by a secondary screening of full texts that met the criteria. In cases of disagreement, resolution was achieved through discussion or adjudication by a third researcher. Data extraction included the following elements: first author, publication year, study region, sample size, age, intervention, control measures, and outcomes. For each included study, we extracted post-intervention values for all pre-specified outcomes (BMI, SBP, DBP, HDL, LDL, TC, TG, and IL-6). For studies with missing or unreported data, we attempted to contact the corresponding authors to obtain the necessary information. If data remained unavailable, standard imputation methods recommended by the Cochrane Handbook, such as estimating means and standard deviations from medians, ranges, or interquartile ranges, were applied. This approach ensures methodological transparency and reproducibility of the meta-analytic estimates.

### 2.4 Risk of bias

The methodological quality of included studies was assessed using the Cochrane Collaboration’s Risk of Bias tool 2.0 (RoB 2.0) (Sterne et al., 2019). This tool assesses the risk of bias in studies across five domains: bias arising from the randomization process, bias arising from deviations in intervention implementation, bias arising from missing outcome data, bias arising from outcome measurement, and bias arising from selective reporting of results. The risk of bias in each domain is rated as “low risk,” “some concern,” or “high risk,” leading to an overall assessment of the study’s risk of bias. Risk of bias assessments were conducted independently by two researchers. Disagreements were resolved through discussion and negotiation, with a third researcher involved for adjudication when necessary.

### 2.5 GRADE evaluation

The quality of evidence for primary outcome measures was assessed using the Grading of Recommendations Assessment, Development and Evaluation (GRADE) approach (Brignardello-Petersen et al., 2023). The GRADE system comprehensively evaluates the overall quality of evidence from meta-analyses across five dimensions: study limitations (risk of bias), inconsistency of results, indirectness, imprecision, and publication bias. Evidence from randomized controlled trials was initially rated as “high” quality, with downgrades applied as appropriate based on the aforementioned factors. Final evidence quality was categorized into four levels: high, moderate, low, or very low. Evidence quality assessments were conducted independently by two researchers, with disagreements resolved through discussion and consensus.

### 2.6 Statistical analysis

This study employed systematic review and meta-analysis methods to integrate metabolic, cardiovascular, and inflammation-related outcomes in adult males undergoing combined aerobic exercise interventions. Considering potential clinical and methodological differences among included studies in subject characteristics, intervention protocols, outcome measurements, and follow-up durations, all meta-analyses adopted random-effects models to yield more robust and conservative effect estimates.

For continuous outcomes, weighted mean differences (WMD) were used because all included studies reported outcomes on the same measurement scale, facilitating clinically interpretable comparisons. Standardized mean differences (SMD) were considered but deemed unnecessary due to scale consistency. Corresponding 95% confidence intervals were reported.

Inter-study heterogeneity was assessed using the I² statistic. Potential sources of heterogeneity were explored through pre-specified subgroup analyses based on participants’ comorbidities, intervention methods, control types, and intervention duration, as well as meta-regression analyses using these factors as covariates. Sensitivity analyses were further conducted by sequentially excluding individual studies to evaluate the robustness of pooled results.

Potential publication bias was visualized using funnel plots and quantitatively assessed with Egger’s regression test. Egger’s regression test was applied to evaluate small-study effects; however, we acknowledge that Egger’s test has limited reliability when fewer than 10 studies are included, and results should be interpreted with caution. All statistical analyses were performed in Stata (Version 15.0; StataCorp, College Station, TX, USA), primarily using the “metan” command for meta-analysis, “metareg” for meta-regression, and standard procedures for subgroup, sensitivity, and publication bias analyses.

## 3 Results

### 3.1 Literature screening results

**Figure 1:**
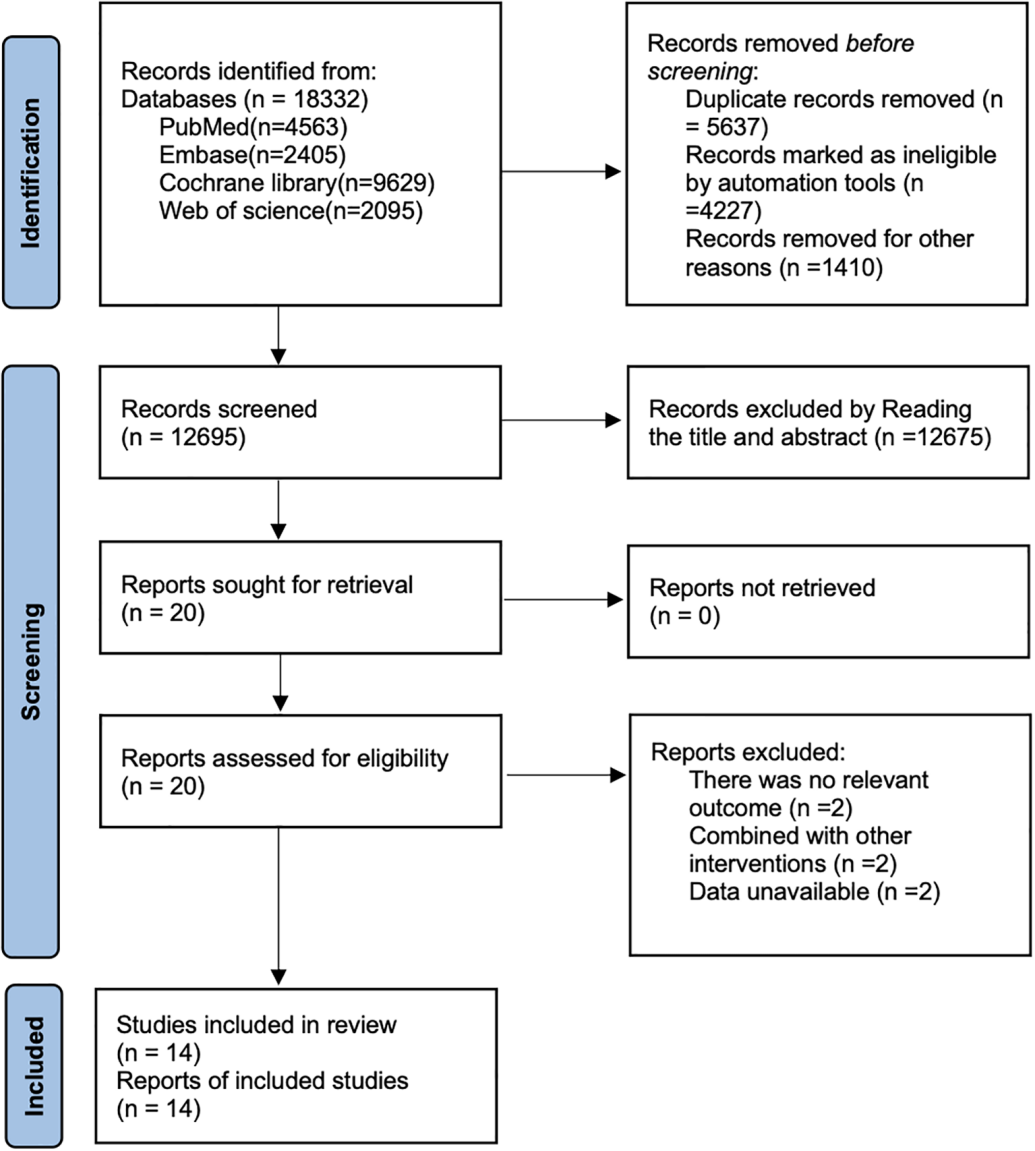
PRISMA flow diagram of study selection.

By searching PubMed (n=4563), Embase (n=2405), Cochrane Library (n=9629), and Web of Science (n=2095), a total of 18,332 articles were retrieved. After removing 5,637 duplicate records, 12,675 articles were excluded based on title and abstract screening, and 6 articles were excluded after full-text review. Ultimately, 14 studies (Galvão et al., 2010; Sousa et al., 2013; Azarbayjani et al., 2014; Zhao and Liang, 2019; Park et al., 2020; Caminiti et al., 2021; Hematinezhad Touli et al., 2022; Mohammadrezaei et al., 2022; Park et al., 2022; Alemayehu and Teferi, 2023; Amare et al., 2024; Acheche et al., 2025; Kumari et al., 2025; Mostaghimi and Heidarianpour, 2025) were included for analysis. The detailed screening process is illustrated in **Figure 1**.

### 3.2 Basic characteristics of included study

This study included 14 randomized controlled trials published between 2010 and 2025, covering regions across Africa, Asia, Europe, and Oceania. A total of 369 adult males were enrolled, with participants ranging in age from approximately 21 to 70 years, encompassing young, middle-aged, and elderly populations. Some subjects had comorbidities including chronic obstructive pulmonary disease, hypertension, overweight/obesity, prostate cancer, or ischemic stroke, while others were healthy individuals without significant comorbidities, indicating a degree of heterogeneity in the included populations. The intervention group primarily employed combined aerobic exercise interventions involving resistance training combined with aerobic exercise. Some studies combined aerobic exercise with measures such as nutritional supplementation, phytochemicals, or electrical stimulation. The control groups mostly received aerobic exercise alone or conventional controls. The most common outcome measure was BMI, followed by blood pressure (SBP, DBP) and lipid profiles (HDL, LDL, TC, TG). Some studies reported IL-6 levels. Detailed characteristics of the included studies are presented in **Table 1**.

**Table 1:** The basic characteristics table of the included literature.

Author	Year	Country	Sample size		Mean age		Comorbidity	Intervention		Outcomes
			EG	CG	EG	CG		EG	CG	
Acheche	2025	Tunisia	16	15	65	67	COPD	resistance and aerobic training	aerobic training	BMI; IL-6
Alemayehu	2023	Ethiopia	12	12	44	47.08	Hypertensive	resistance and aerobic training group	control group	BMI; SBP; DBP
Amare	2024	Ethiopia	7	7	48.71	49.83	Overweight/obesity	resistance and aerobic training	aerobic training	HDL; LDL; TC
Azarbayjani	2014	New Zealand	10	10	22.9	22.9	NO	resistance and aerobic training	control group	BMI; HDL; LDL; TG; TC
Caminiti	2021	Italy	28	27	69	67.6	Hypertensive	resistance and aerobic training	aerobic training	SBP; DBP
Galvao	2010	Australia	29	28	69.5	70.1	Prostate Cancer	resistance and aerobic training	control group	HDL; LDL; TC; TG
Hematinezhad	2022	Iran	10	10	32	32	Overweight/obesity	green tea and aerobic training	control group	BMI; SBP; DBP; HDL; LDL; TC
Kumari	2025	India	10	10	22	22	Overweight/obesity	resistance and aerobic training	aerobic training	BMI
Mohammadrezaei	2022	Iran	10	10	23.75	25.91	NO	hemp seed powder and aerobic training	control group	HDL; LDL; TC; TG
Mostaghimi	2025	Iran	8	8	67.63	68.88	Ischemic stroke	electrical stimulation and aerobic training	control group	IL-6; HDL; LDL
Park	2022	Korea	8	8	64.63	64.25	NO	Phytoncide and aerobic training	control group	BMI
Park	2020	Korea	10	10	69.1	68.5	Overweight/obesity	resistance and aerobic training	control group	IL-6; BMI; SBP; DBP; HDL; LDL; TC; TG
Sousa	2013	Portugal	17	15	62.1	53.2	NO	resistance and aerobic training	control group	BMI; SBP; DBP
Zhao	2019	China	12	12	21.43	21.45	Overweight/obesity	resistance and aerobic training	control group	BMI

EG, Experimental group; CG, Control group; COPD, chronic obstructive pulmonary disease; BMI, body mass index, SBP, systolic blood pressure, DBP, diastolic blood pressure; HDL, High-density lipoprotein cholesterol, LDL, Low-density lipoprotein, TC, Total cholesterol; TG, Triglyceride; IL-6, Interleukin-.

### 3.3 Risk of bias results

This study employed ROB 2.0 for quality assessment. Results (**Supplementary Materials**, **Supplementary Figures 1, 2**) indicate that two included studies were rated as “some concerns” due to unclear reporting of the randomization scheme used. All other studies were rated as low risk. Overall, the included studies were of high quality. GRADE results (**Supplementary Material**, **Supplementary Table 2**) indicate that BMI, SBP, DBP, HDL, TC, TG and LDL were of moderate quality, while IL-6 were low-quality outcomes.

### 3.5 Meta-analysis results

#### 3.5.1 BMI

**Figure 2:**
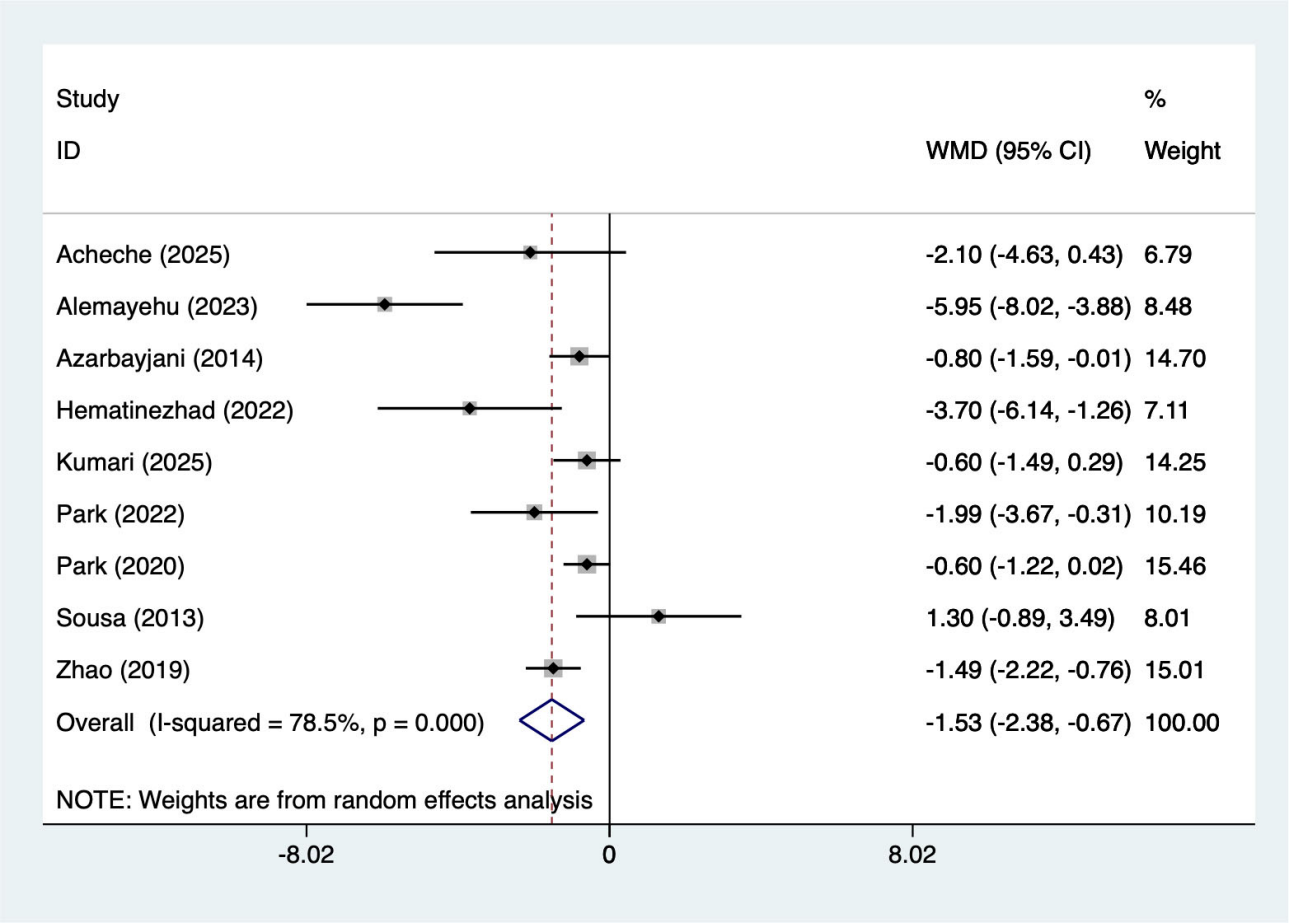
Meta-analysis forest plot of BMI.

9 studies reported BMI (207 participants), with heterogeneity testing (I²=78.5%, P = 0.001). Analysis using a random-effects model (**Figure 2**) indicated that combined interventions based on aerobic exercise may reduce BMI in adult males (WMD=-1.53, 95%CI [-2.38 to -0.67]). Due to significant heterogeneity, sensitivity analysis (**Supplementary Material**, **Supplementary Figure 3**) confirmed stable results unaffected by individual studies.

#### 3.5.2 HDL

**Figure 3:**
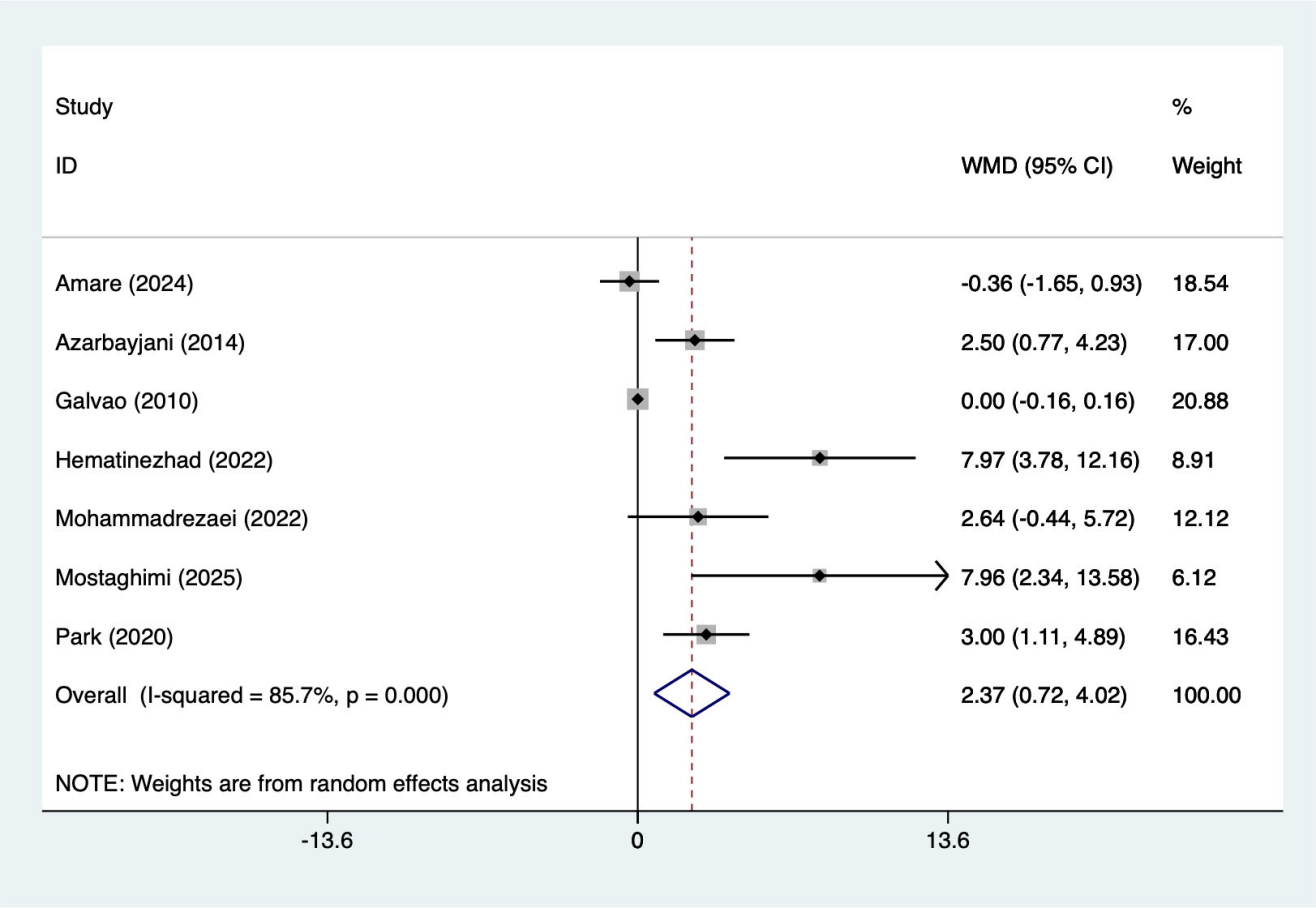
Meta-analysis forest plot of HDL.

7 studies reported HDL (167 participants), with heterogeneity testing (I²=85.7%, P = 0.001). Analysis using a random-effects model (**Figure 3**) indicated that combined interventions based on aerobic exercise may increase HDL in adult males (WMD = 2.37, 95%CI [0.72 to 4.02]). Due to significant heterogeneity, sensitivity analysis (**Supplementary Material**, **Supplementary Figure 4**) confirmed stable results unaffected by individual studies.

#### 3.5.3 LDL

**Figure 4:**
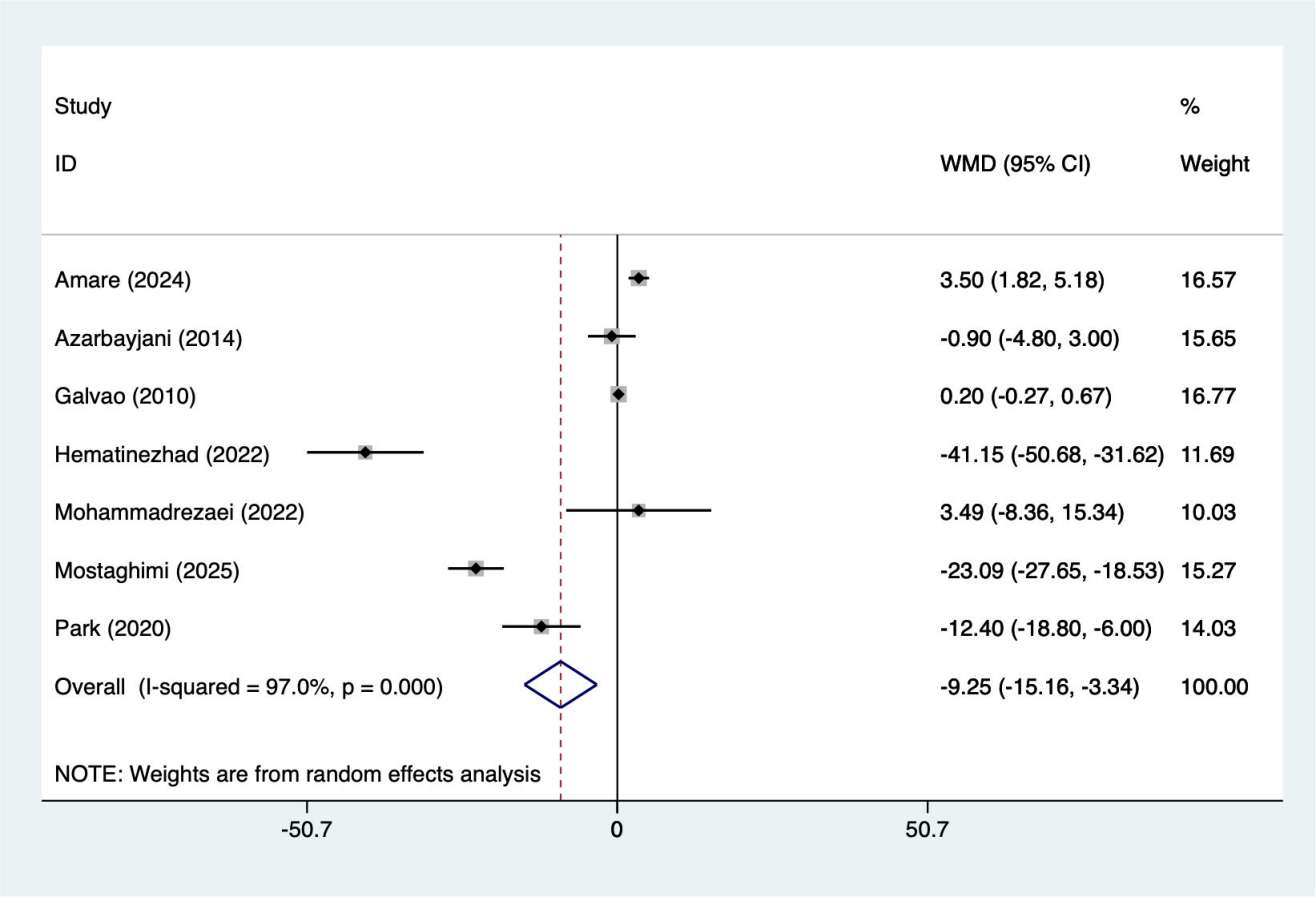
Meta-analysis forest plot of LDL.

7 studies reported LDL (167 participants), with heterogeneity testing (I²=97.0%, P = 0.001). Analysis using a random-effects model (**Figure 4**) indicated that combined interventions based on aerobic exercise may reduce LDL in adult males (WMD=-9.25, 95%CI [-15.16 to -3.34]). Due to significant heterogeneity, sensitivity analysis (**Supplementary Material**, **Supplementary Figure 5**) confirmed stable results unaffected by individual studies.

#### 3.5.4 TC

**Figure 5:**
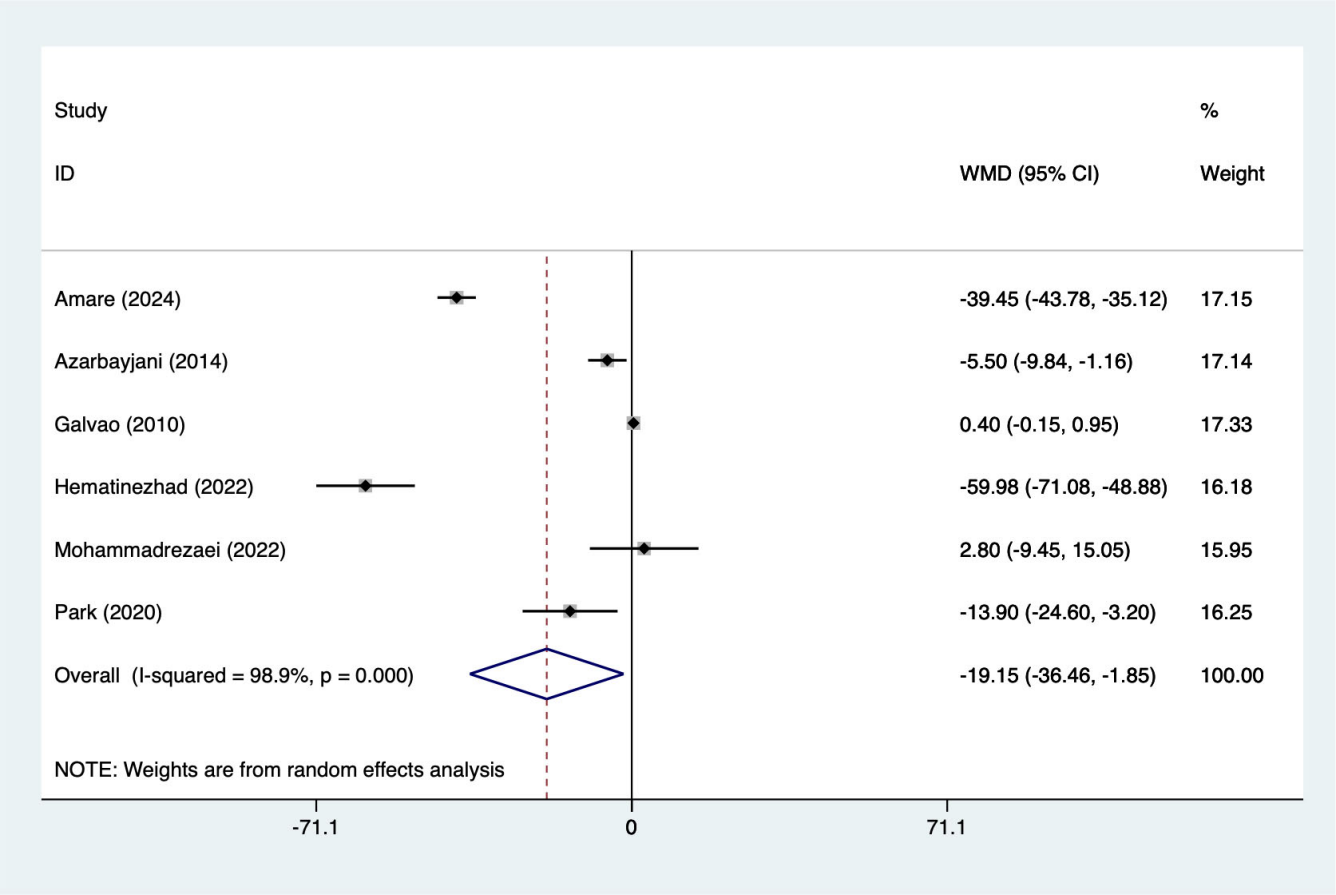
Meta-analysis forest plot of TC.

6 studies reported TC (151 participants), with heterogeneity testing (I²=98.9%, P = 0.001). Analysis using a random-effects model (**Figure 5**) indicated that combined interventions based on aerobic exercise may reduce TC in adult males (WMD=-19.15, 95%CI [-36.46 to -1.85]). Due to significant heterogeneity, sensitivity analysis (**Supplementary Material**, **Supplementary Figure 6**) confirmed stable results unaffected by individual studies.

#### 3.5.5 TG

**Figure 6:**
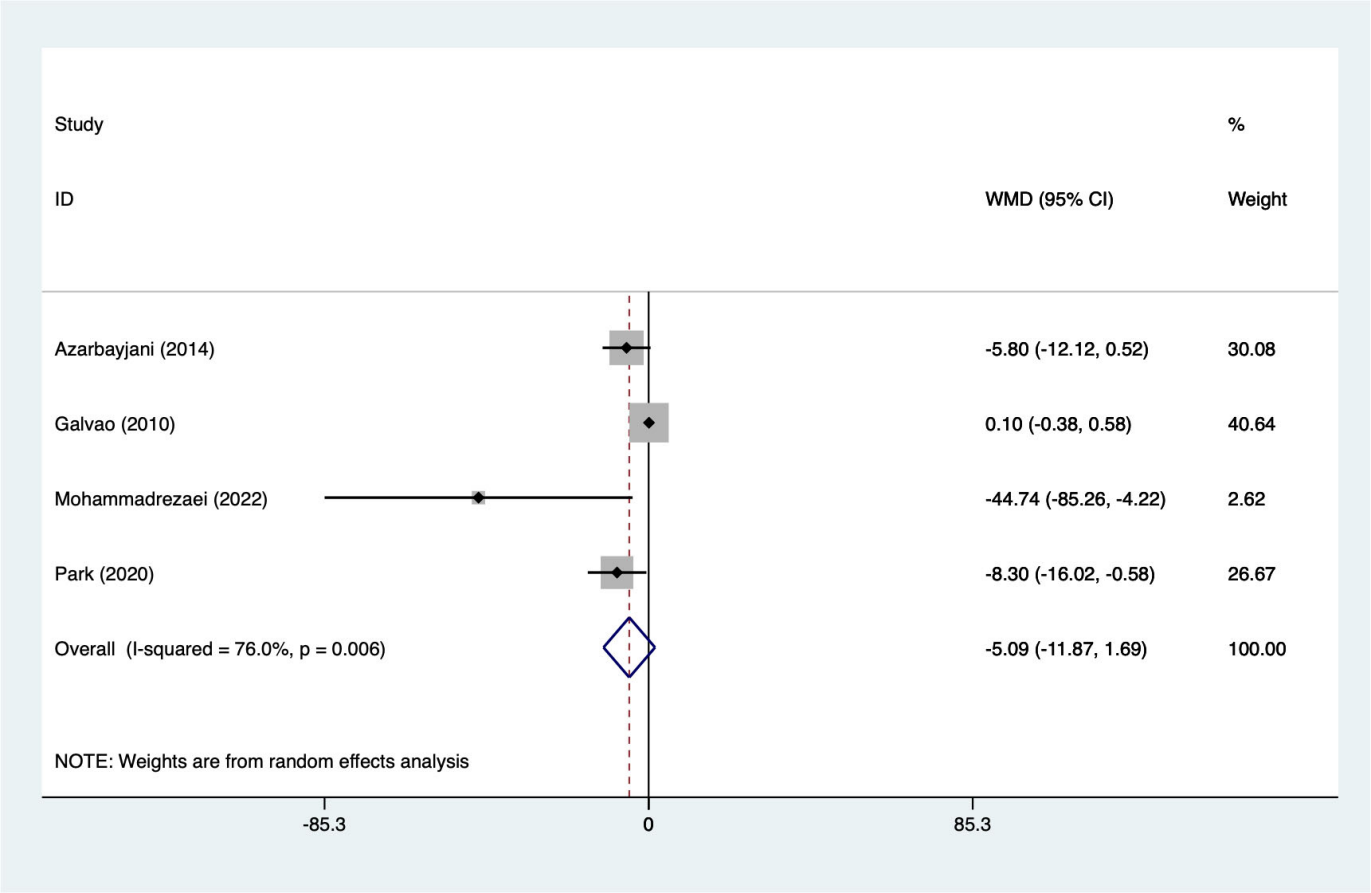
Meta-analysis forest plot of TG.

4 studies reported on TG (117 participants). Heterogeneity testing (I² = 76.0%, P = 0.006). Analysis using a random-effects model (**Figure 6**) showed a non-significant reduction in TG levels following combined aerobic exercise-based interventions (WMD=-5.09, 95%CI [-11.87 to 1.69]). Although the effect estimate suggests a trend toward reduction, the confidence interval crossed zero. Given the limited number of included studies and the presence of considerable heterogeneity, these findings should be interpreted with caution. Sensitivity analysis (**Supplementary Material**, **Supplementary Figure 7**) confirmed stable results unaffected by individual studies.

#### 3.5.6 IL-6

**Figure 7:**
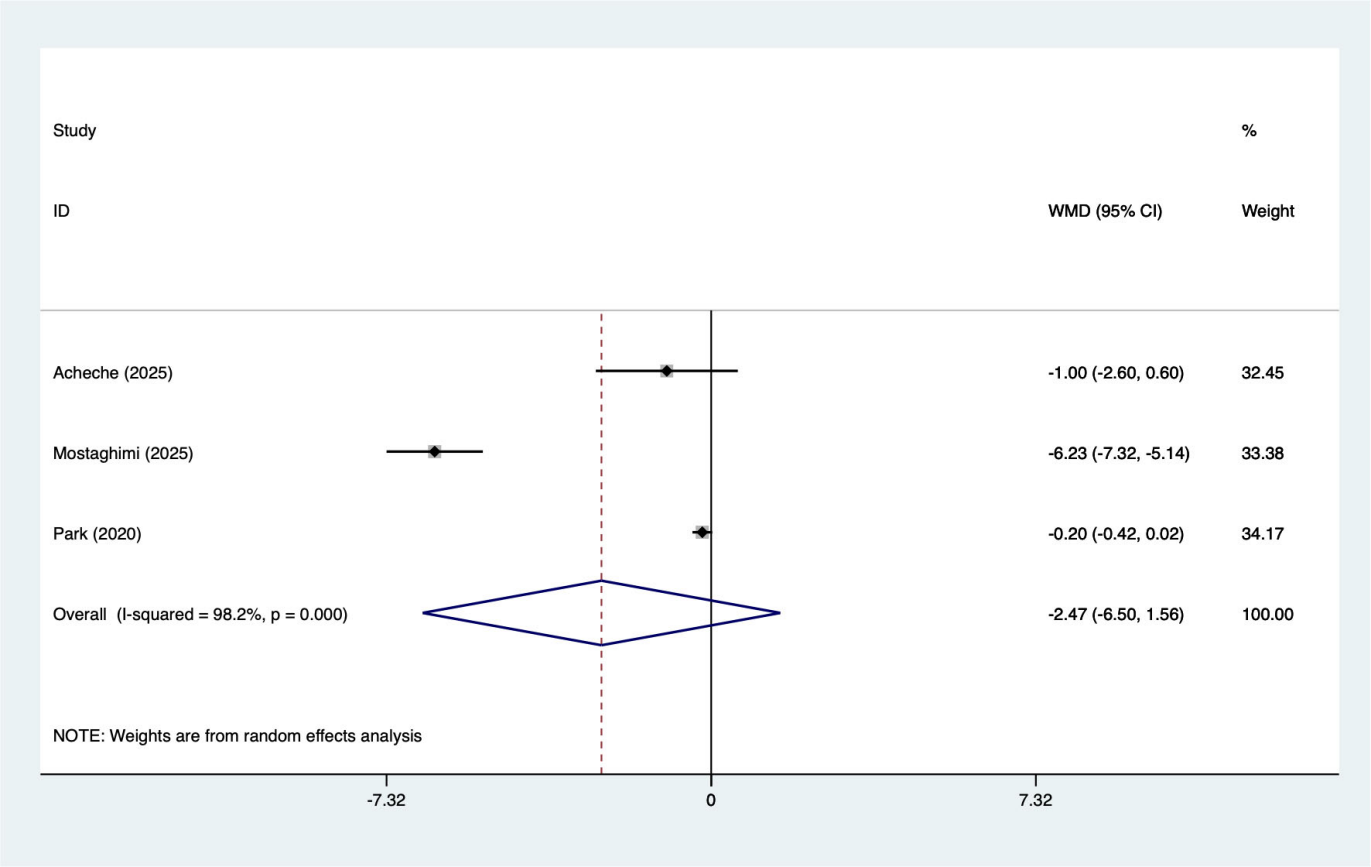
Meta-analysis forest plot of IL-6.

3 studies reported on IL-6 (67 participants). Heterogeneity testing (I² = 98.2%, P = 0.006). Analysis using a random-effects model (**Figure 7**) suggested a non-significant decrease in IL-6 levels (WMD = −2.47, 95% CI [−6.50 to 1.56]). The direction of effect indicates a potential reduction; however, the confidence interval included zero and statistical power is limited due to the very small number of studies. Sensitivity analysis (**Supplementary Material**, **Supplementary Figure 8**) confirmed stable results unaffected by individual studies.

#### 3.5.7 SBP

**Figure 8:**
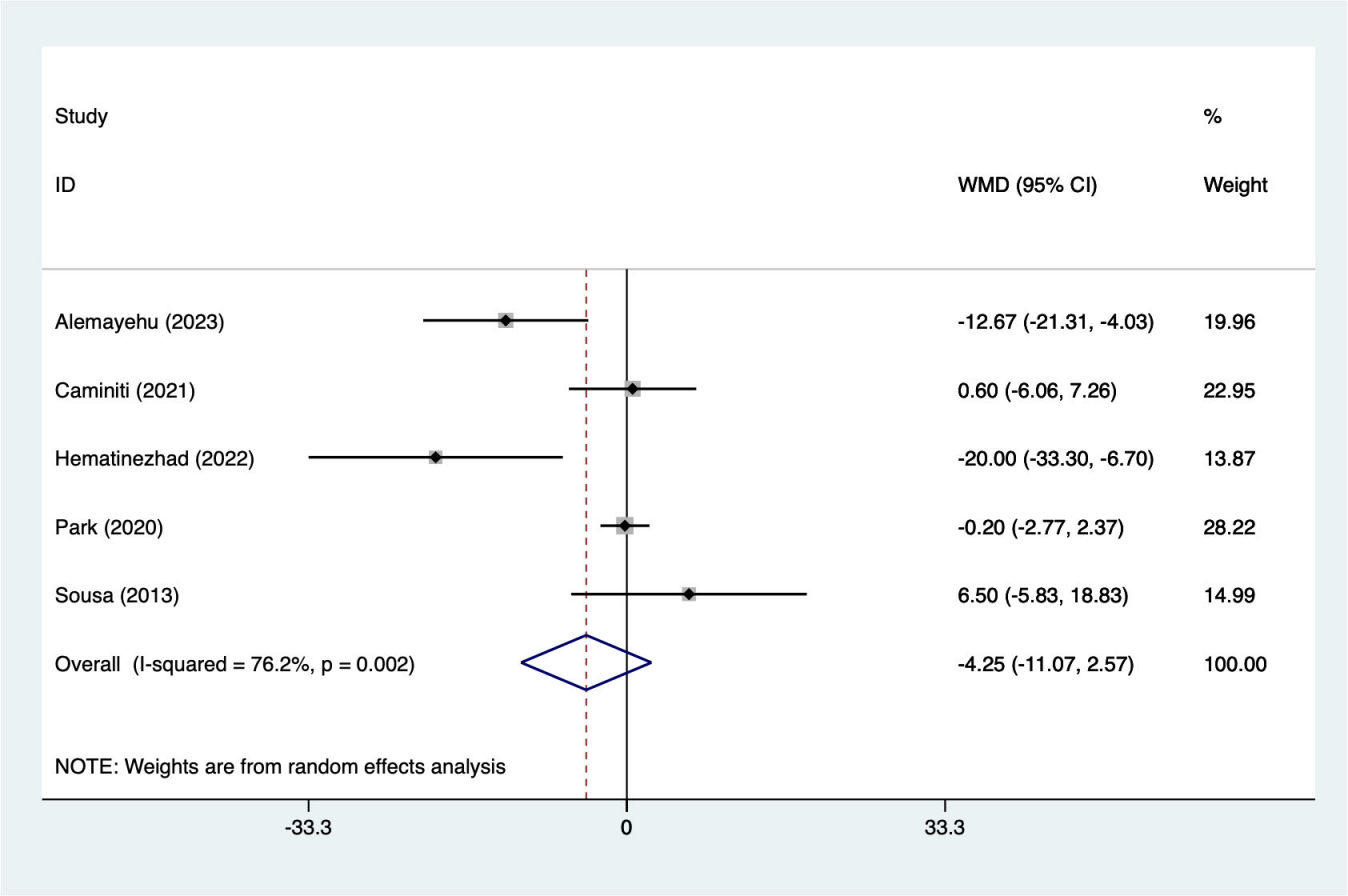
Meta-analysis forest plot of SBP.

5 studies reported on SBP (151 participants). Heterogeneity testing (I² = 76.2%, P = 0.002). Analysis using a random-effects model (**Figure 8**) indicated a non-significant reduction in SBP (WMD = −4.25, 95% CI [−11.7 to 2.57]). While the pooled estimate suggests a trend toward lower SBP, the wide confidence interval crossing zero indicates statistical non-significance. Considering the limited number of included studies and the extremely high heterogeneity, the robustness of this pooled estimate is uncertain. sensitivity analysis (**Supplementary Material**, **Supplementary Figure 9**) confirmed stable results unaffected by individual studies.

#### 3.5.8 DBP

**Figure 9:**
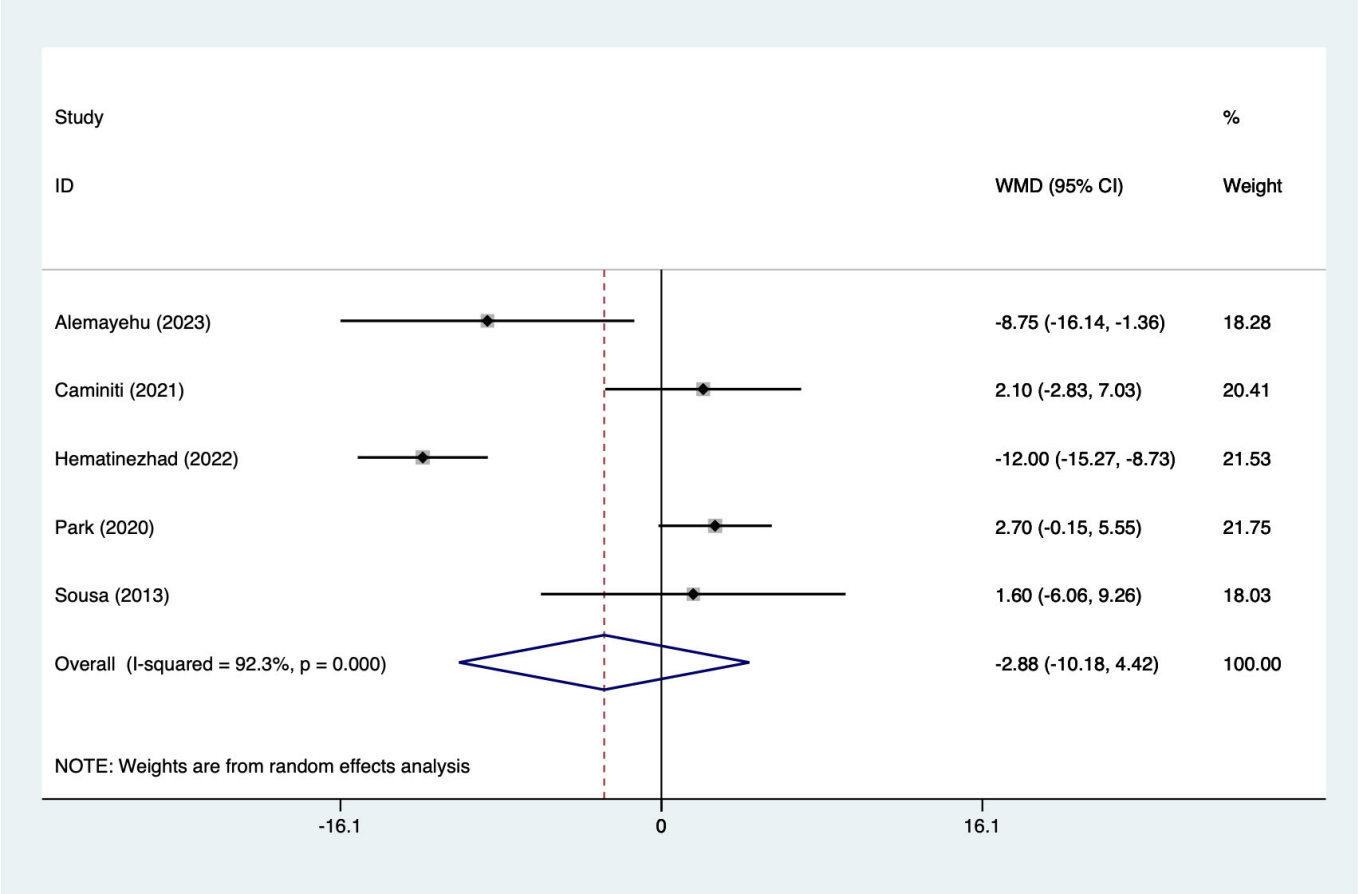
Meta-analysis forest plot of DBP.

5 studies reported on DBP (151 participants). Heterogeneity testing (I² = 92.3%, P = 0.001). Analysis using a random-effects model (**Figure 9**) indicated a non-significant reduction DBP (WMD=-2.88, 95%CI [-10.18 to 4.42]). While the pooled estimate suggests a trend toward lower DBP, the wide confidence interval crossing zero indicates statistical non-significance. Given the relatively small number of studies and high between-study variability, this finding should be interpreted cautiously. Sensitivity analysis (**Supplementary Material**, **Supplementary Figure 10**) confirmed stable results unaffected by individual studies.

### 3.6 Subgroup analysis

Subgroup analyses were conducted based on intervention type, comorbidities, control group, and intervention duration (**Table 2**).

**Table 2:** subgroup analysis results.

Outcomes	Group	Subgroup	No of study	Heterogeneity (%)	WMD 95%CI
BMI	Comorbidity	YES	6	83.8	-2.04(-3.20, -0.87)
		NO	3	63.5	-0.67 (-2.10, 0.76)
	Intervention	Resistance and aerobic training	7	80.9	-1.28 (-2.20, -0.36)
		green tea and aerobic training	1	NA	-3.7 (-6.14, -1.26)
		Phytoncide and aerobic training	1	NA	-1.99 (-3.67, -0.31)
	Control	Control	7	83	-1.69 (-2.75, -0.62)
		aerobic training	2	16.5	-0.86 (-1.97, 0.25)
	Week	8week	4	70.6	-1.14 (-2.9, 0.62)
		12week	5	84.8	-1.81(-2.91, -0.70)
HDL	Comorbidity	YES	5	87.3	2.36 (0.32, 4.4)
		NO	2	0	-0.67 (-2.10, 0.76)
	Intervention	Resistance and aerobic training	4	83.1	1.07 (-0.35, 2.49)
		green tea and aerobic training	1	NA	7.97 (3.78, 12.16)
		hemp seed powder and aerobic training	1	NA	2.64 (-0.44, 5.72)
		electrical stimulation and aerobic training	1	NA	7.96 (2.34, 13.58)
	Control	Control	6	87.9	3.27 (1.00, 5.54)
		aerobic training	1	NA	-0.39 (-1.65, 0.93)
	Week	8week	4	86.3	4.07 (-0.18, 8.31)
		12week	3	88.5	1.69 (-0.53, 3.90)
LDL	Comorbidity	YES	5	98	-12.95 (-20.32, -5.99)
		NO	2	0	-0.67 (-2.10, 0.76)
	Intervention	Resistance and aerobic training	4	89.9	-0.87 (-4.13, 2.38)
		green tea and aerobic training	1	NA	-41.15 (-50.68, -31.62)
		hemp seed powder and aerobic training	1	NA	3.49 (-8.36, 15.34)
		electrical stimulation and aerobic training	1	NA	-23.09 (-27.65, -18.53)
	Control	Control	6	97.3	3.5 (1.82, 5.18)
		aerobic training	1	NA	-23.09 (-27.65, -18.53)
	Week	8week	4	98.4	-14.29 (-34.54, 5.96)
		12week	3	86.7	-3.45 (-8,.91, 2.01)
TC	Comorbidity	YES	4	99.3	-28.04 (-56.37, 0.29)
		NO	2	36.2	-3.41 (-10.47, 3.66)
	Intervention	Resistance and aerobic training	4	99.1	-14.5(-33.9, 4.79)
		green tea and aerobic training	1	NA	-59.8 (-71.08, -48.88)
		hemp seed powder and aerobic training	1	NA	2.80 (-9.45, 15.05)
	Control	Control	5	96.8	-14.65 (-28.00, -1.29)
		aerobic training	1	16.5	-39.45 (-43.78, -35.12)
	Week	8week	3	96.6	-32.45 (-60.52, -4.37)
		12week	3	85.5	-4.61 (-11.1, 1.88)
TG	Comorbidity	YES	2	78	-3.18 (-11.21, 4.85)
		NO	2	71.1	-19.9 (-56.6, 16.77)
	Intervention	Resistance and aerobic training	3	74.4	-3.72 (-9.40, 1.96)
		hemp seed powder and aerobic training	1	NA	-44.74 (-85.62, -4.22)
	Week	8week	3	74.4	-3.72 (-9.40, 1.96)
		12week	1	NA	-44.74 (-85.62, -4.22)
SBP	Comorbidity	YES	4	80.3	-6.25 (-13.91, 1.41)
		NO	1	NA	6.50 (-5.83, 18.83)
	Intervention	Resistance and aerobic training	4	66.5	-1.70 (-7.51, 4.12)
		green tea and aerobic training	1	NA	-20.0 (-33.3, -6.7)
	Control	Control	4	81.8	-6.04 (-15.82, 3.73)
		aerobic training	1	NA	0.60 (-6.06, 7.26)
	Week	8week	3	73.7	-3.13 (-9.41, 3.83)
		12week	2	87.8	-6.63 (-32.6, 19.34)
DBP	Comorbidity	YES	4	94.1	-3.81 (-12.33, 4.58)
		NO	1	NA	1.60 (-6.06, 9.26)
	Intervention	Resistance and aerobic training	4	63.1	0.16 (-4.21, 4.53)
		green tea and aerobic training	1	NA	-12.0 (-15.27, -8.73)
	Control	Control	4	93.7	2.10 (-2.83, 7.03)
		aerobic training	1	NA	0.60 (-6.06, 7.26)
	Week	8week	3	75.4	-0.42 (-6.04, 5.20)
		12week	2	90.2	-5.66 (-18.96, 7.64)
IL-6	Intervention	Resistance and aerobic training	2	0	-0.22 (-0.44, 0.01)
		electrical stimulation and aerobic training	1	NA	-6.23 (-7.32, -5.14)
	Control	Control	2	99.1	-3.19 (-9.10, 2.72)
		aerobic training	1	NA	-1.0 (-2.60, 0.60)
	Week	8week	2	96.4	-3.65 (-8.77, 1.48)
		12week	1	0	-0.20 (-0.42, 0.02)

BMI, body mass index; SBP, systolic blood pressure; DBP, diastolic blood pressure; HDL, High-density lipoprotein cholesterol; LDL, Low-density lipoprotein; TC, Total cholesterol; TG, Triglyceride; IL-6, Interleukin-6; NA, Not applicable; WMD, Weighted mean difference.

For BMI, reductions were observed among participants with underlying conditions (6 studies, WMD = −2.04, 95% CI −3.20 to −0.87) and in interventions of approximately 12 weeks (5 studies, WMD = −1.81, 95% CI −2.91 to −0.70). Resistance training combined with aerobic exercise (7 studies, WMD = −1.28, 95% CI −2.20 to −0.36) was also associated with lower BMI.

In contrast, findings for green tea combined with aerobic exercise (1 study, WMD = −3.70, 95% CI −6.14 to −1.26) and plant essence combined with aerobic exercise (1 study, WMD = −1.99, 95% CI −3.67 to −0.31) were derived from single studies. These results should be considered exploratory and not equivalent to pooled estimates.

For lipid outcomes, HDL appeared higher in participants with comorbidities (5 studies, WMD = 2.36, 95% CI 0.32 to 4.40), while LDL was lower in the same subgroup (5 studies, WMD = −12.95, 95% CI −20.32 to −5.99). However, apparent effects observed in specific intervention subgroups, such as green tea or electrical stimulation combined with aerobic exercise, were based on individual studies and should not be interpreted as consistent evidence.

For TC, reductions were observed in the control group comparison subgroup (5 studies, WMD = −14.65, 95% CI −28.00 to −1.29) and in shorter-duration interventions (3 studies, WMD = −32.45, 95% CI −60.52 to −4.37). Notably, large effect sizes reported in certain subgroups (e.g., green tea) were based on single studies.

For TG, apparent reductions were observed in the 12-week subgroup and in the flaxseed powder subgroup; however, both findings were derived from single studies and should be interpreted cautiously.

Similarly, improvements in SBP, DBP, and IL-6 observed in specific subgroups were based on single-study evidence and do not represent pooled meta-analytic findings.

### 3.7 Meta regression

Meta-regression (**Table 3**) analyses were performed to explore potential sources of between-study heterogeneity across outcomes. Comparator type was associated with variation in BMI effect estimates (β = 0.85, P = 0.01), suggesting that differences in control conditions may partially influence the observed effect sizes. No statistically significant associations were observed for other covariates, including country, publication year, intervention type, or comorbidity status across the evaluated outcomes (all P > 0.05). Several variables, such as publication year in BMI (P = 0.06) and SBP (P = 0.07), showed borderline trends but did not reach statistical significance.

**Table 3:** Meta-regression analysis of potential sources of heterogeneity across outcomes.

Variable	BMI (Coefficient, P)	HDL (Coefficient, P)	LDL (Coefficient, P)	TC (Coefficient, P)	TG (Coefficient, P)	IL-6 (Coefficient, P)	SBP (Coefficient, P)	DBP (Coefficient, P)
Country	0.86, 0.77	0.96, 0.34	0.18, 0.07	0.13, 0.37	0.97, 0.13	-0.37, 0.65	-0.26, 0.75	-0.34, 0.28
Year	-0.46, 0.06	0.86, 0.47	-0.24, 0.81	0.34, 0.85	0.24, 0.76	-0.49,0.85	0.26, 0.07	-0.08, 0.13
Intervention	0.13. 0.24	0.54, 0.76	-0.34, 0.26	-0.48, 0.75	0.57, 0.86	-0.24, 0.86	0.27, 0.86	0.31, 0.96
Control	0.85, 0.01	0.75, 0.29	0.27, 0.58	-0.43, 0.16	0.13, 0.81	0.63, 0.28	-0.43. 0.61	0.09, 0.12
Comorbidity	-0.24, 0.09	-0.34, 0.73	0.86, 0.38	0.64, 0.19	0.37, 0.26	0.75, 0.14	0.23, 0.66	0.86, 0.96

Given the limited number of included studies and the exploratory nature of meta-regression, these findings should be interpreted with caution. Residual heterogeneity remained substantial, indicating that additional unmeasured factors may contribute to variability across studies.

### 3.8 Publication bias

Publication bias was assessed using funnel plots and Egger’s regression test (**Supplementary Figures 11**–**18**). For BMI (P = 0.179), SBP (P = 0.382), DBP (P = 0.971), LDL (P = 0.173), TC (P = 0.134), and IL-6 (P = 0.481), Egger’s test did not indicate statistically significant asymmetry. However, evidence of potential small-study effects was observed for HDL (P = 0.013) and TG (P = 0.002), suggesting possible publication bias for these outcomes. Visual inspection of funnel plots also indicated asymmetry for these measures.

It should be noted that most outcomes included fewer than 10 studies, limiting the statistical power and reliability of Egger’s test. Therefore, these findings should be interpreted cautiously and considered suggestive rather than definitive evidence of publication bias.

## 4 Discussion

### 4.1 Main finding

Overall, this meta-analysis suggests that combined aerobic exercise-based interventions may be associated with reductions in BMI and changes in certain lipid parameters (HDL, LDL, and TC) in adult males. However, the magnitude of these effects was modest, and their clinical relevance remains uncertain in the absence of established minimal important differences or long-term outcome data.

However, given the substantial heterogeneity observed across most outcomes and the limited number of studies for several indicators, these findings should be interpreted cautiously. According to the GRADE assessment, the certainty of evidence was rated as moderate for BMI, SBP, DBP, HDL, TC, TG, and LDL, and low for IL-6. Moderate-quality evidence suggests that the true effect is likely close to the estimated effect, although further research may still influence the confidence in the estimate. In contrast, the low certainty rating for IL-6 indicates that the true effect may be substantially different from the pooled estimate. Therefore, while the findings for anthropometric, lipid, and blood pressure outcomes suggest potential changes, conclusions should remain cautious—particularly for inflammatory markers, where the evidence base is limited and less certain.

Subgroup analyses indicated variation in BMI across different conditions, including individuals with comorbidities and approximately 12-week interventions. Certain multimodal approaches, such as aerobic exercise combined with resistance training or specific nutritional/physical therapies, showed larger effect estimates in some analyses. Nevertheless, several subgroup findings were derived from single studies and therefore should be considered exploratory rather than confirmatory. Many subgroup findings were based on limited data or single studies and should therefore be interpreted as exploratory.

Although sensitivity analyses suggested that pooled estimates were not driven by individual studies, the extremely high heterogeneity for outcomes such as LDL, TC, IL-6, and DBP indicates that effects were not consistently observed across all trials. Therefore, the present findings should be regarded as indicative of potential trends rather than definitive evidence of efficacy.

### 4.2 Mechanism comparison

First, regarding body composition, this study found that combined aerobic intervention significantly reduced BMI, with more pronounced effects observed in individuals with comorbidities and during the 12-week intervention period. These findings suggest that intervention duration and participants’ health status may be critical factors influencing weight changes (Bucher et al., 2014). Individuals with underlying conditions typically exhibit higher levels of metabolic abnormalities, thus presenting greater potential for improvement through exercise interventions (Prior et al., 2014). Furthermore, the 12-week intervention demonstrated more stable outcomes, indicating that short-term exercise may be insufficient to produce significant weight changes (Cho et al., 2024). A 12-week intervention are more conducive to establishing a stable energy deficit, thereby promoting body fat reduction. Combined interventions incorporating resistance training, aerobic exercise plus green tea or plant extracts demonstrated favorable outcomes, suggesting that multimodal approaches may synergistically enhance weight loss through multiple pathways—including increased energy expenditure, improved insulin sensitivity, and enhanced lipid oxidation (Feng et al., 2025).

Regarding lipid profiles, this study found significant increases in HDL and decreases in LDL and TC, consistent with previous exercise intervention studies. Mechanistically, aerobic exercise enhances lipoprotein lipase activity to promote triglyceride breakdown while improving cholesterol reverse transport, thereby elevating HDL levels and reducing LDL and TC (Wood et al., 2023). Subgroup analyses revealed more pronounced improvements in HDL and LDL levels with green tea combined with exercise and electrical stimulation combined with exercise. This may be attributed to catechins in green tea promoting lipid metabolism and enhancing fat oxidation rates, as well as electrical stimulation increasing muscle activation levels (Guo et al., 2024; Mouskeftara et al., 2024). However, some subgroups included only a single study, and the stability of these results requires further validation.

Notably, this study found no overall significant effect of combined aerobic interventions on TG, potentially due to the limited number of included studies, small sample sizes, and substantial individual dietary variations. Subgroup analysis revealed a significant downward trend in TG levels in the flaxseed powder combined exercise or 12-week intervention groups, suggesting dietary components and intervention duration may be critical factors influencing TG changes (Wang et al., 2026). Future studies should rigorously control dietary factors to clarify the true impact of combined exercise interventions on TG.

Regarding inflammatory markers, the overall meta-analysis did not show a significant reduction in IL-6, but a significant improvement was observed in the electrical stimulation combined with aerobic training subgroup. As an inflammatory marker with strong short-term exercise responsiveness, IL-6 levels are highly influenced by exercise intensity, frequency, and blood sampling timing, leading to substantial inter-study variability (Gomarasca et al., 2022; Kim and Rhyu, 2025). Furthermore, only three studies reported IL-6 data, and their high heterogeneity contributed significantly to the lack of overall statistical significance. Future high-quality RCTs are needed to validate the long-term effects of combined interventions on inflammatory markers.

The blood pressure results indicate that combined aerobic intervention did not achieve statistically significant differences for either SBP or DBP overall. However, the effect direction showed a downward trend for both parameters (SBP: WMD = -4.25 mmHg; DBP: WMD = -2.88 mmHg), suggesting potential cardiovascular benefits. Polyphenols in green tea may exert antihypertensive effects by improving vascular endothelial function, reducing oxidative stress, and enhancing nitric oxide production (Poon et al., 2024). Meanwhile, electrical stimulation or resistance training increases muscle pump function and peripheral vascular compliance, potentially amplifying exercise’s regulatory impact on blood pressure (Lopes et al., 2023). Subgroup analyses suggested potentially greater blood pressure–lowering trends in certain combination patterns (green tea combined with aerobic training). However, these findings were based on a limited number of studies with small sample sizes and should therefore be interpreted as exploratory rather than confirmatory. Future large-scale, long-term follow-up studies are needed to further validate the real-world efficacy of combined aerobic interventions in blood pressure management.

### 4.3 Sensitivity discussion

This study also observed high statistical heterogeneity (I² > 75% for most indicators), potentially stemming from multiple sources: First, variations existed in intervention methods, encompassing diverse combinations such as resistance training, nutritional supplementation, and electrical stimulation. Second, study populations differed in health status, including both healthy individuals and those with metabolic abnormalities or chronic diseases. Third, discrepancies were present in intervention duration, exercise intensity, and frequency. These factors may all influence intervention outcomes, suggesting that findings should be interpreted with caution.

### 4.4 Heterogeneity and interpretation

A central methodological feature of this meta-analysis is the substantial statistical heterogeneity observed across most outcomes (I² frequently exceeding 75%, and in some cases approaching 90–99%). Such high heterogeneity indicates that the magnitude and direction of effects varied considerably between studies.

Several potential sources may explain this variability. Intervention heterogeneity was substantial, with included studies evaluating diverse combined models. Intervention duration, intensity, and participant characteristics also varied markedly across studies.

Although subgroup and meta-regression analyses were conducted to explore potential sources of heterogeneity, only comparator type showed an association with BMI estimates, and most variability remained unexplained. This suggests that additional unmeasured factors may contribute to between-study differences.

Therefore, pooled estimates should be interpreted as average effects across heterogeneous settings rather than as precise or universally applicable effect sizes.

### 4.5 Clinical significance

The findings of this study suggest that aerobic exercise–based combined interventions may be associated with changes in weight status and lipid parameters in adult males. However, given the modest effect sizes, high heterogeneity, and lack of long-term outcome data, the clinical significance of these findings remains uncertain. These results should therefore not be interpreted as evidence supporting the superiority of combined interventions, but rather as indicating potential benefits that may vary depending on specific intervention contexts.

### 4.6 Strengths and limitations

This study’s strengths lie in its systematic inclusion of multiple combined aerobic intervention models and comprehensive assessment of multidimensional outcomes. However, several limitations exist. First, most outcome measures exhibited high heterogeneity, and only a limited proportion of this variability could be explained by subgroup or meta-regression analyses. Second, many included interventions were multimodal, and the observed effects cannot be attributed solely to aerobic exercise itself. Third, some subgroups comprised only a single study, limiting the strength of evidence. Finally, the overall sample size was small, and follow-up durations were relatively short, limiting the assessment of long-term outcomes.

### 4.7 Future research directions

Future studies should conduct large-sample, multicenter randomized controlled trials with standardized intervention intensity, frequency, and duration to reduce inter-study heterogeneity and enhance evidence quality. Given that several included studies involved participants with comorbid conditions (obesity, hypertension, metabolic disorders), future research should also stratify analyses by baseline health status to determine whether intervention effects differ across risk profiles. Additionally, more research is needed to compare the relative efficacy of different combined intervention models (exercise + diet, exercise + nutritional supplementation, exercise + physical therapy) to identify optimal intervention strategies. Extending follow-up periods and incorporating clinical endpoints (cardiovascular events, metabolic syndrome incidence) will further clarify long-term clinical value and underlying mechanisms.

### 4.8 Conceptual attribution of effects

An important conceptual consideration is that many included interventions were multimodal in nature. Therefore, the observed effects cannot be attributed solely to aerobic exercise itself but rather reflect the combined influence of multiple components. This limits the ability to isolate the independent contribution of aerobic exercise and reduces the interpretability of pooled estimates. Accordingly, the findings should be understood as reflecting the potential effects of integrated intervention strategies rather than aerobic exercise alone.

## 5 Conclusion

This meta-analysis suggests that aerobic exercise–based combined interventions are associated with modest changes in BMI and selected lipid parameters (HDL, LDL, and TC) in adult men. However, no statistically significant overall effects were observed for TG, SBP, DBP, or IL-6, and substantial heterogeneity was present across most outcomes. Given the variability in intervention components, the small number of studies in several analyses, and the inability to isolate the effects of aerobic exercise from other modalities, the findings should be interpreted with caution.

Overall, the current evidence remains limited and does not allow firm conclusions regarding the clinical effectiveness or comparative value of combined aerobic interventions. Further well-designed studies are required to clarify the consistency, magnitude, and clinical relevance of these effects.

## Data Availability

The original contributions presented in the study are included in the article/**Supplementary Material**, further inquiries can be directed to the corresponding author.
