# Supplementary material for: Comparative effects of combined aerobic exercise-based interventions on metabolic, cardiovascular, and inflammatory markers in adult men: a systematic review and meta-analysis
Source: Front Physiol. 2026 Apr 16;17:1810357. doi: 10.3389/fphys.2026.1810357 (PMC13128381; doi:10.3389/fphys.2026.1810357)
Supplement: Supplementary file 1 [file DataSheet1.docx]

Table S1 Literature search strategy

(((("Exercise"[Mesh]) OR (((((Exercise[Title/Abstract]) OR (Exercise, Aerobic[Title/Abstract])) OR (Aerobic Exercise[Title/Abstract])) OR (Aerobic Exercises[Title/Abstract])) OR (Exercises, Aerobic[Title/Abstract]))) AND (("Male"[Mesh]) OR (((Male[Title/Abstract]) OR (Males[Title/Abstract])) OR (Men[Title/Abstract])))) AND (Combined[Title/Abstract])) AND (randomized controlled trial[Publication Type] OR randomized[Title/Abstract] OR placebo[Title/Abstract])

Table S2 GARDE results

| **Certainty assessment** | | | | | | | **№ of patients** | | **Effect** | | **Certainty** | **Importance** |
| --- | --- | --- | --- | --- | --- | --- | --- | --- | --- | --- | --- | --- |
| **№ of studies** | **Study design** | **Risk of bias** | **Inconsistency** | **Indirectness** | **Imprecision** | **Other considerations** | **intervention** | **control** | **Relative (95% CI)** | **Absolute (95% CI)** |  |  |
| **body mass index** | | | | | | | | | | | | |
| 9 | randomised trials | not serious | not serious | not serious | serious | none | 105 | 102 | - | MD **1.53 SD lower** (2.38 lower to 0.67 lower) | ⨁⨁⨁◯ Moderate | CRITICAL |
| **systolic blood pressure** | | | | | | | | | | | | |
| 5 | randomised trials | not serious | not serious | serious | not serious | publication bias strongly suspected all plausible residual confounding would reduce the demonstrated effect | 77 | 74 | - | MD **4.25 lower** (11.07 lower to 2.57 higher) | ⨁⨁⨁◯ Moderate | CRITICAL |
| **diastolic blood pressure** | | | | | | | | | | | | |
| 5 | randomised trials | serious | not serious | not serious | not serious | none | 77 | 74 | - | MD **2.88 lower** (10.82 lower to 4.42 higher) | ⨁⨁⨁◯ Moderate | CRITICAL |
| **high-density lipoprotein** | | | | | | | | | | | | |
| 7 | randomised trials | not serious | serious | not serious | not serious | none | 88 | 84 | - | MD **2.37 higher** (0.72 higher to 4.02 higher) | ⨁⨁⨁◯ Moderate | CRITICAL |
| **low-density lipoprotein** | | | | | | | | | | | | |
| 7 | randomised trials | serious | not serious | not serious | not serious | none | 88 | 84 | - | MD **9.25 lower** (15.16 lower to 3.34 lower) | ⨁⨁⨁◯ Moderate | CRITICAL |
| **total cholesterol** | | | | | | | | | | | | |
| 6 | randomised trials | not serious | not serious | not serious | serious | none | 76 | 75 | - | MD **19.15 lower** (36.46 lower to 1.85 lower) | ⨁⨁⨁◯ Moderate | CRITICAL |
| **triglycerides** | | | | | | | | | | | | |
| 4 | randomised trials | serious | not serious | not serious | not serious | none | 59 | 58 | - | MD **5.09 lower** (11.87 lower to 1.69 higher) | ⨁⨁⨁◯ Moderate | IMPORTANT |
| **Interleukin-6** | | | | | | | | | | | | |
| 3 | randomised trials | serious | not serious | serious | not serious | none | 34 | 33 | - | MD **2.47 lower** (6.5 lower to 1.56 higher) | ⨁⨁◯◯ Low | IMPORTANT |


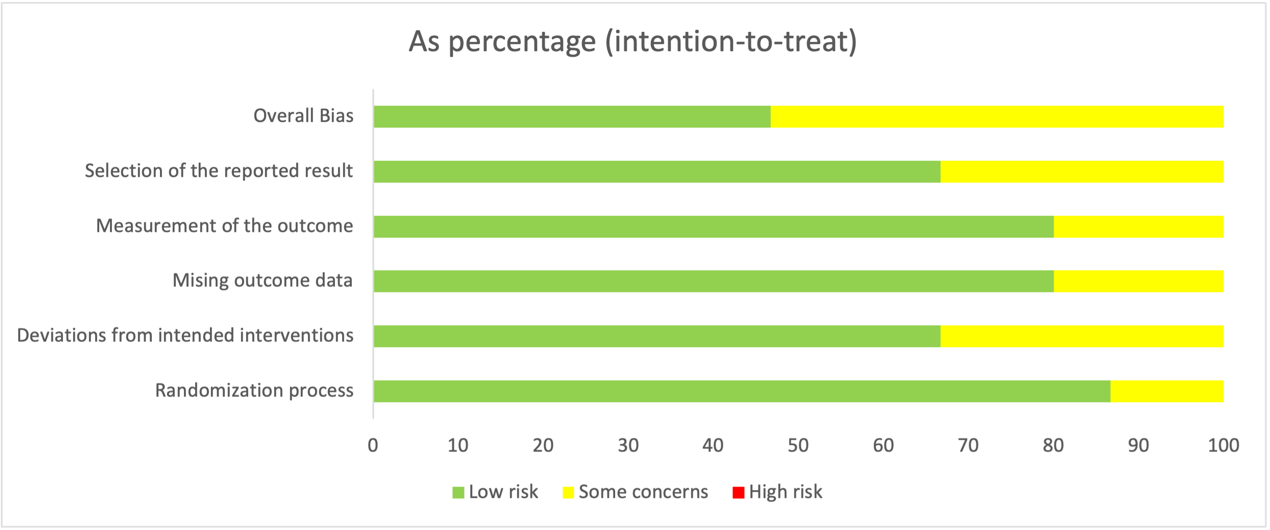


Figure S1 risk of bias summary


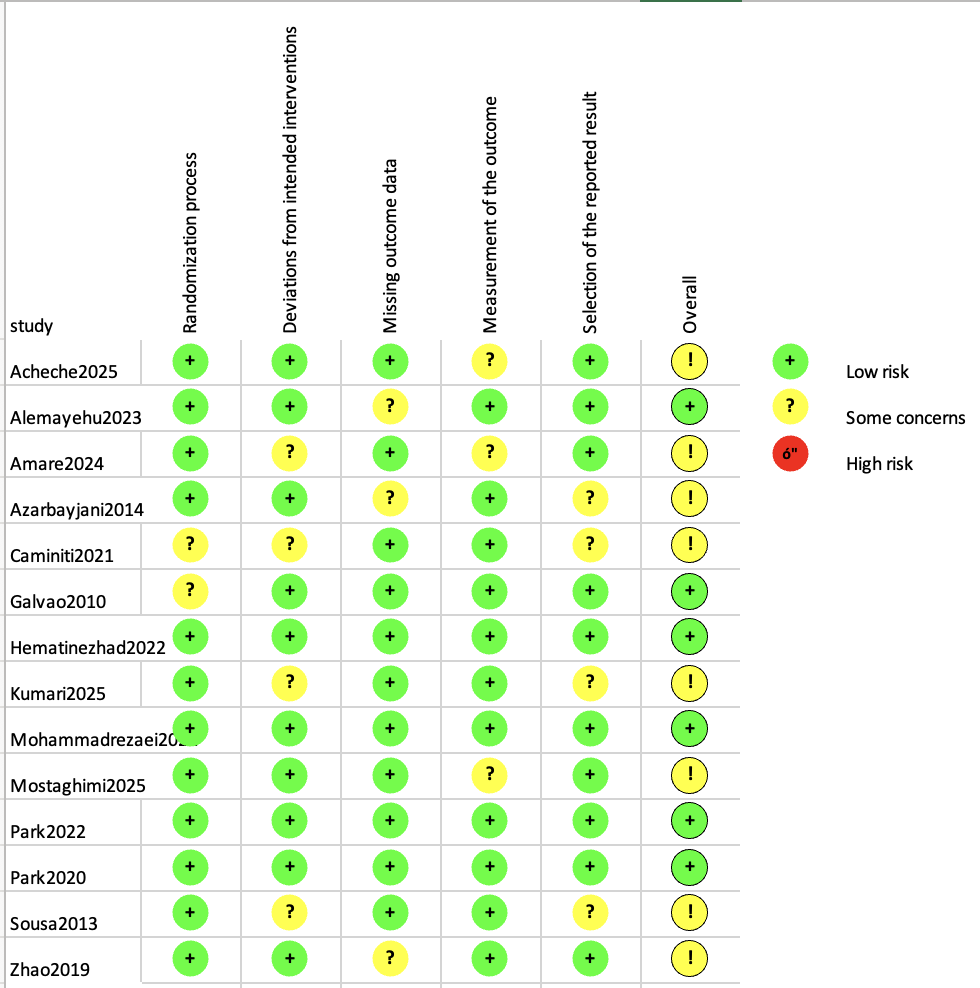


Figure S2 risk of bias graph


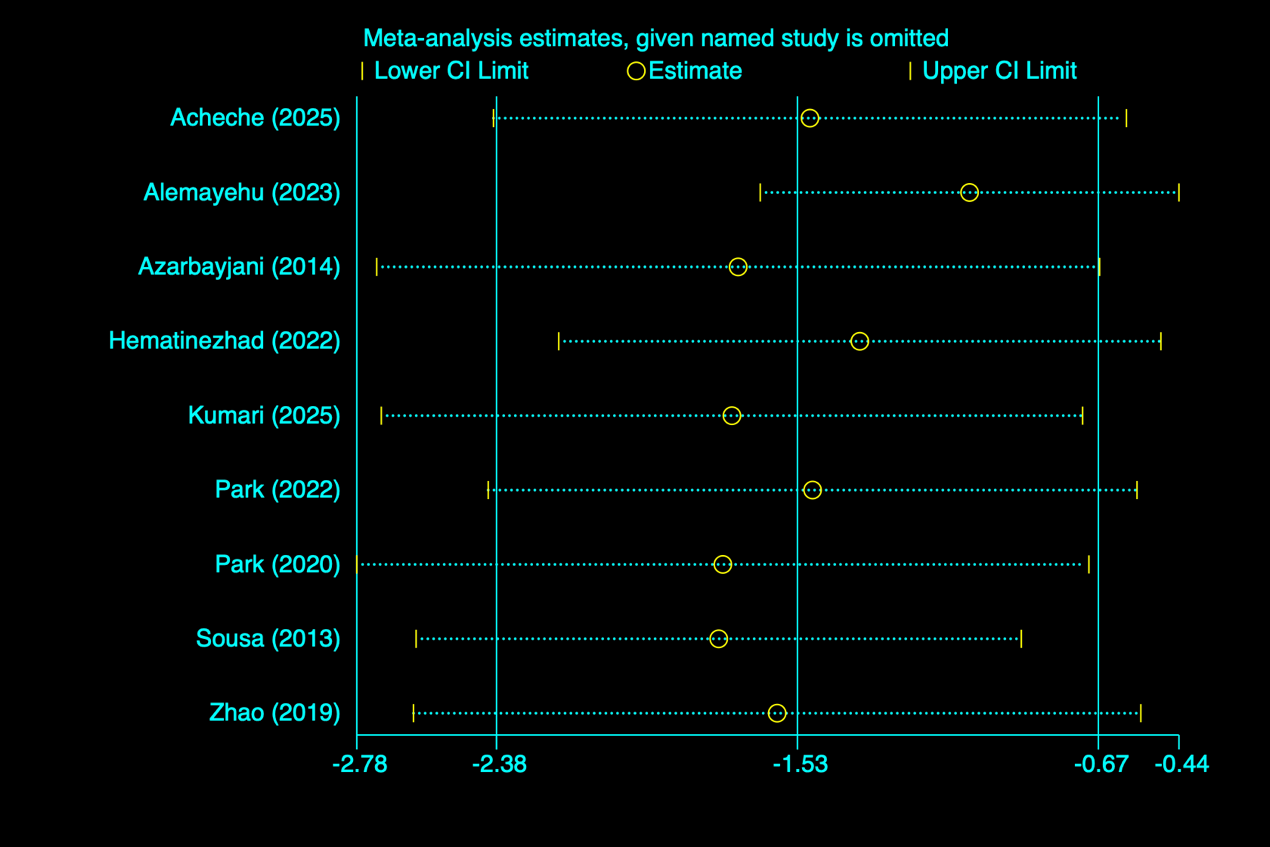


Figure S3 Sensitivity analysis of BMI


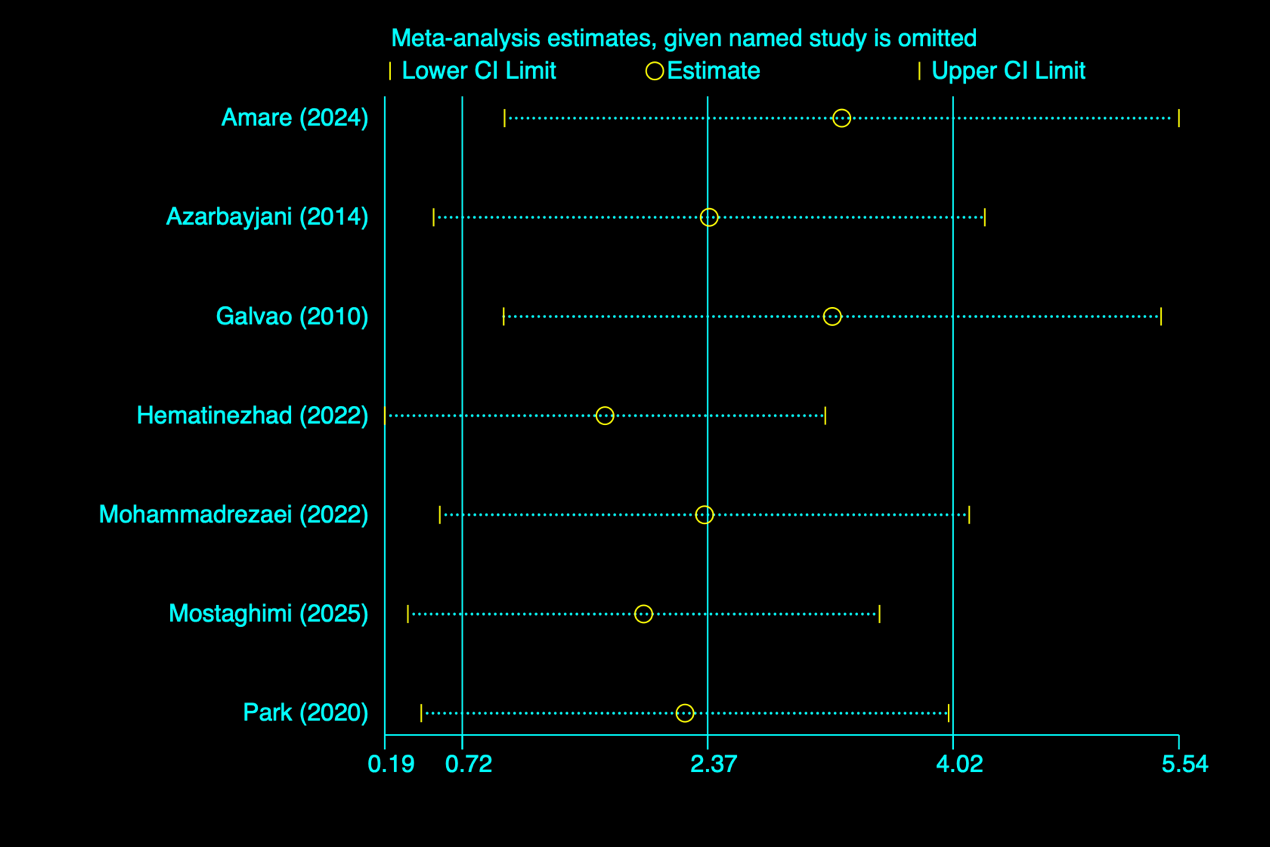


Figure S4 Sensitivity analysis of HDL


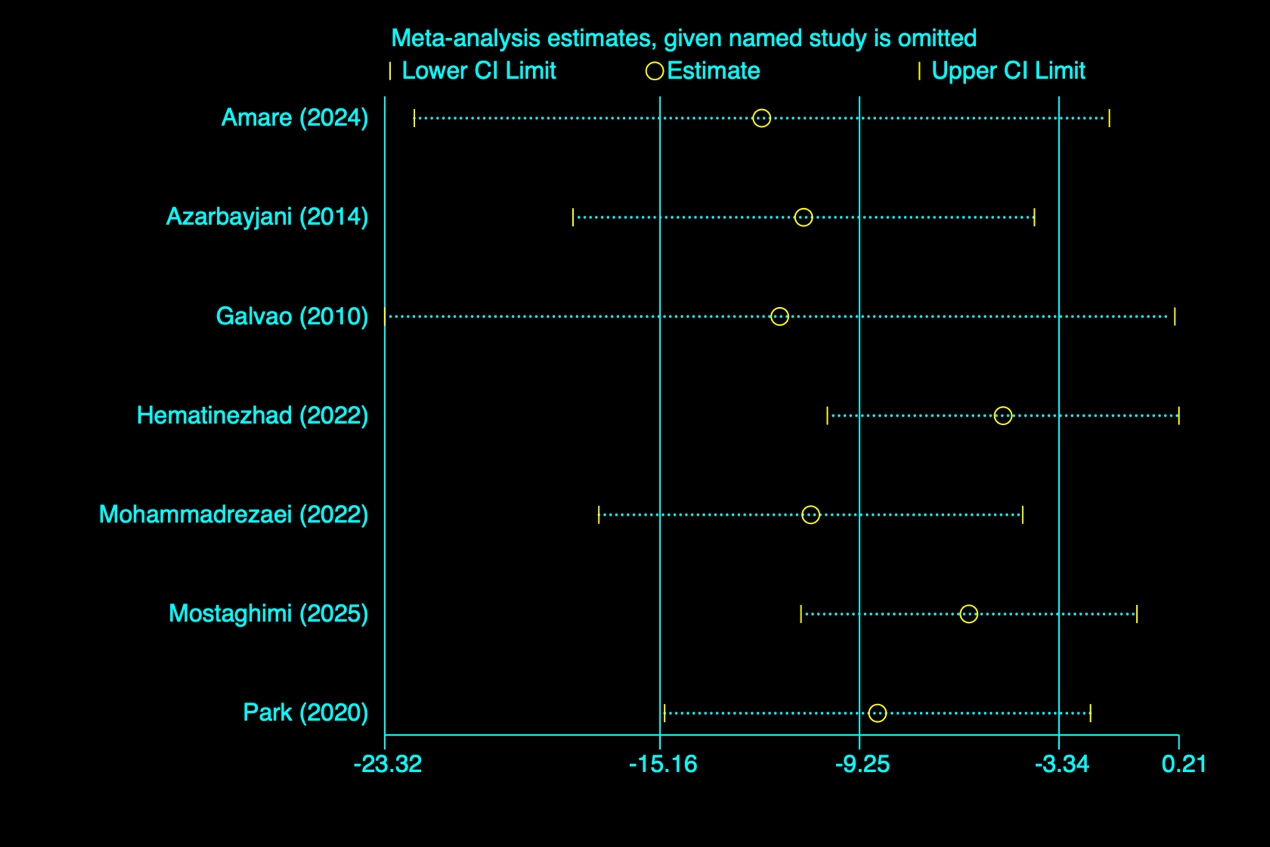


Figure S5 Sensitivity analysis of LDL


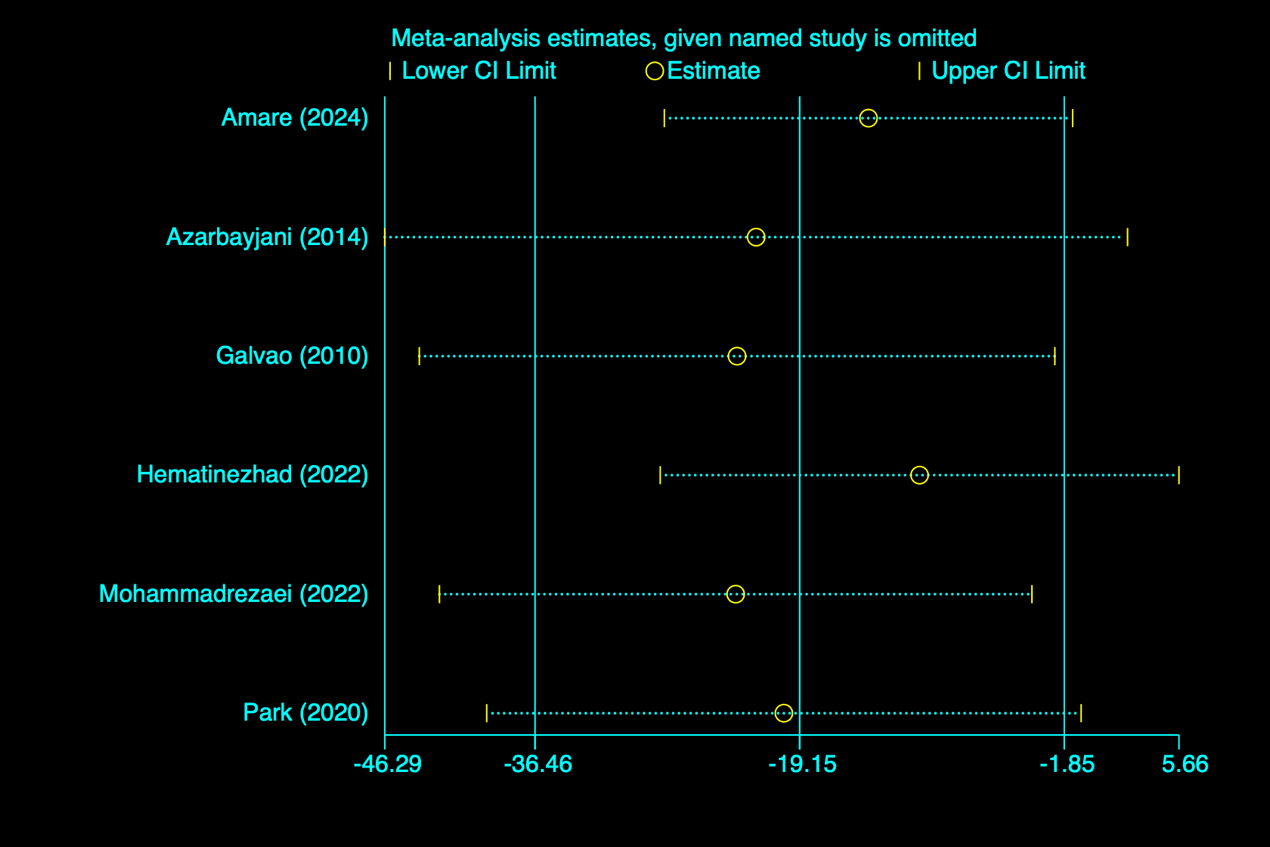


Figure S6 Sensitivity analysis of TC


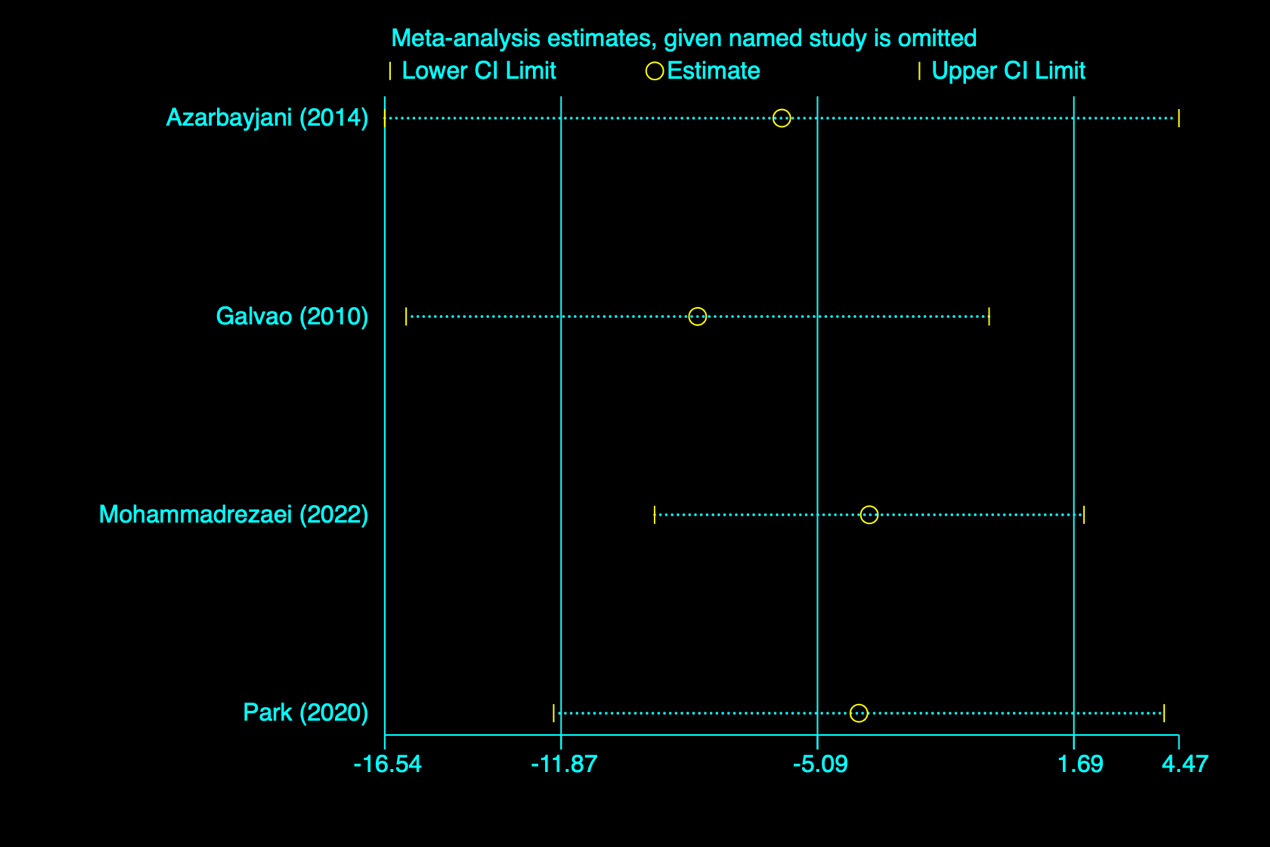


Figure S7 Sensitivity analysis of TG


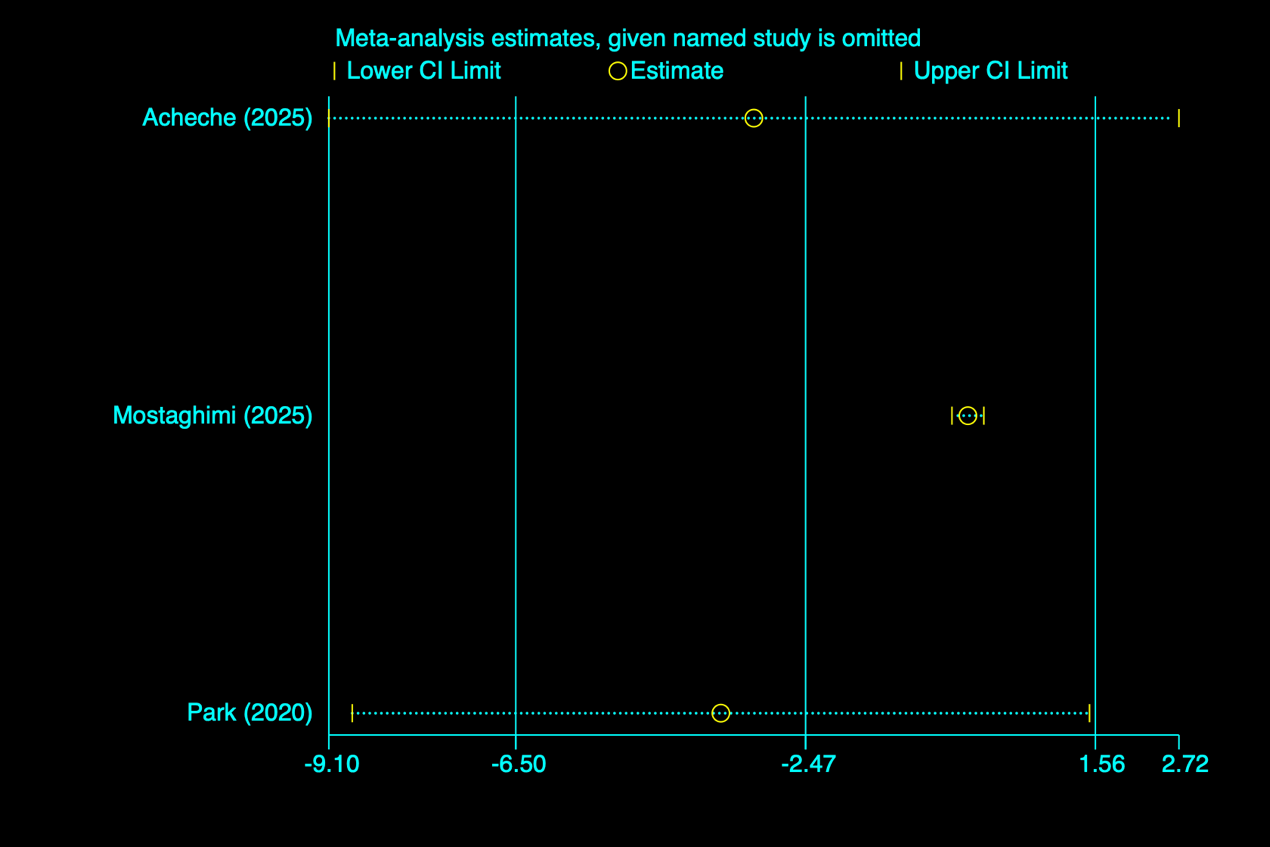


Figure S8 Sensitivity analysis of IL-6


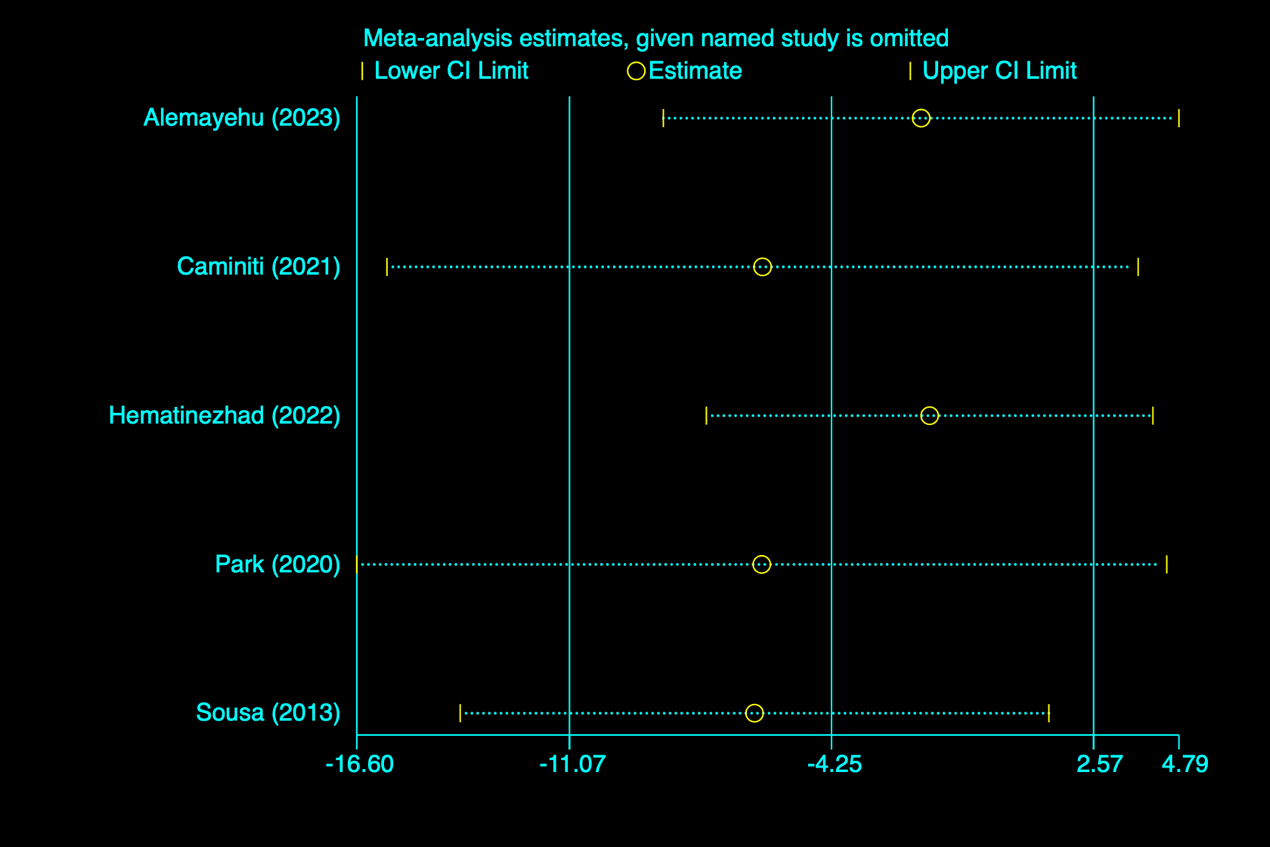


Figure S9 Sensitivity analysis of SBP


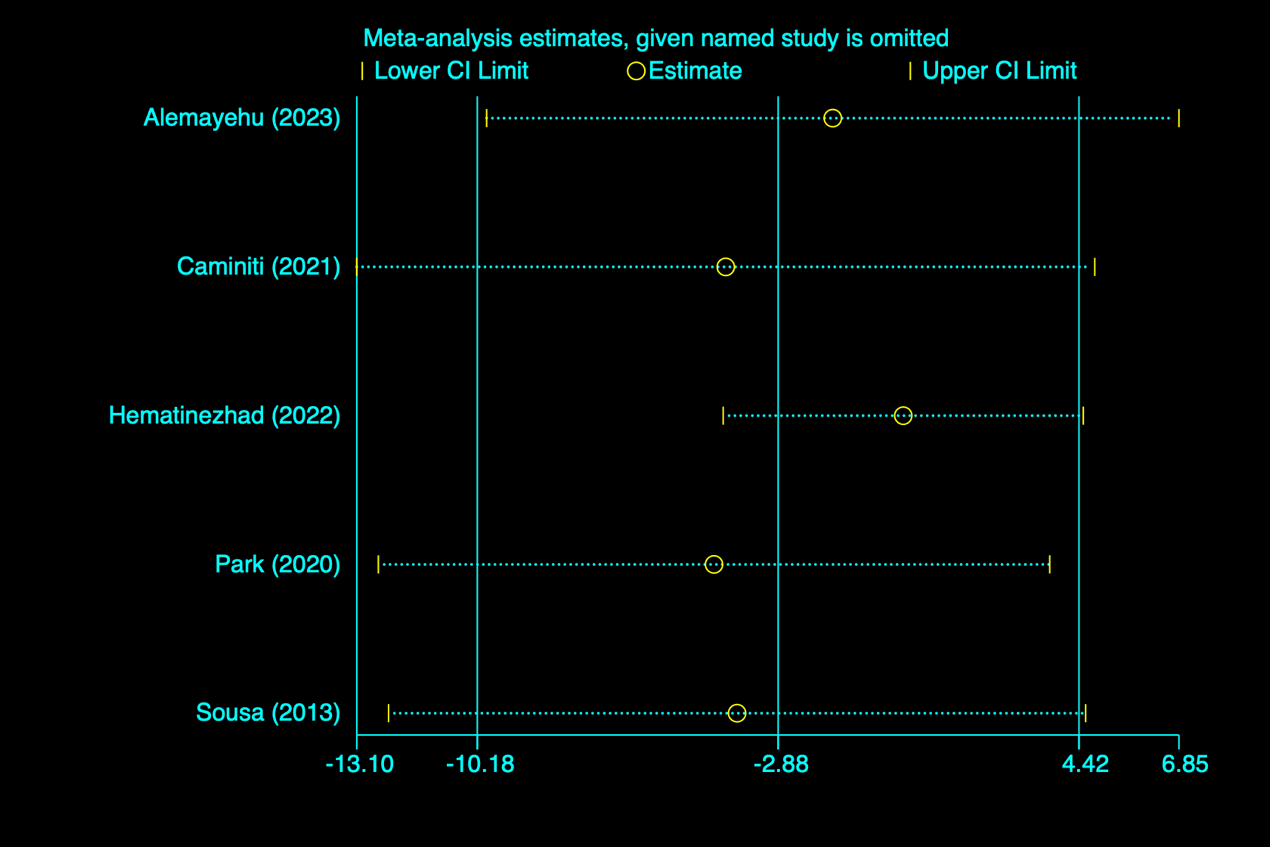


Figure S10 Sensitivity analysis of DBP


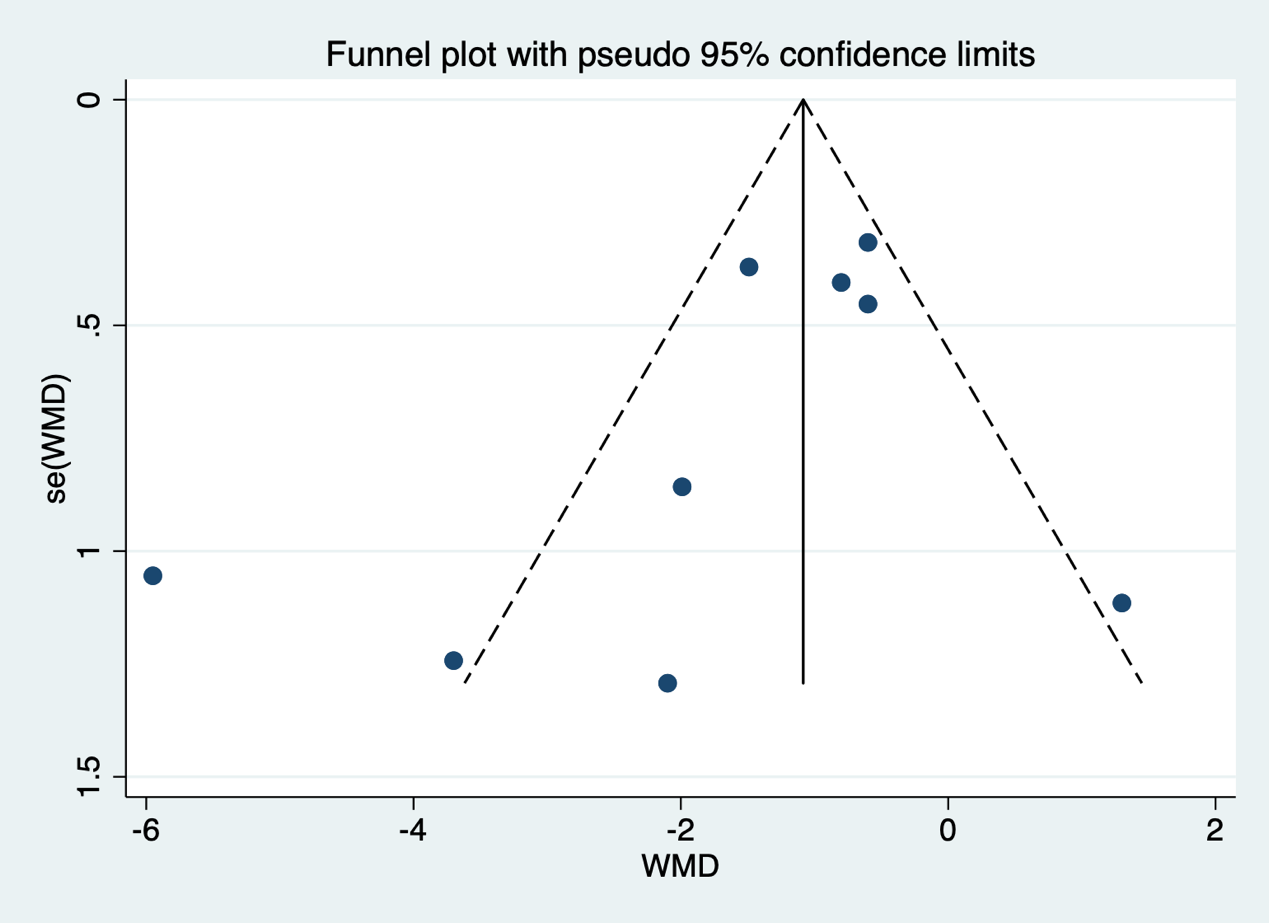


Figure S11 Funnel plot of the meta-analysis of BMI


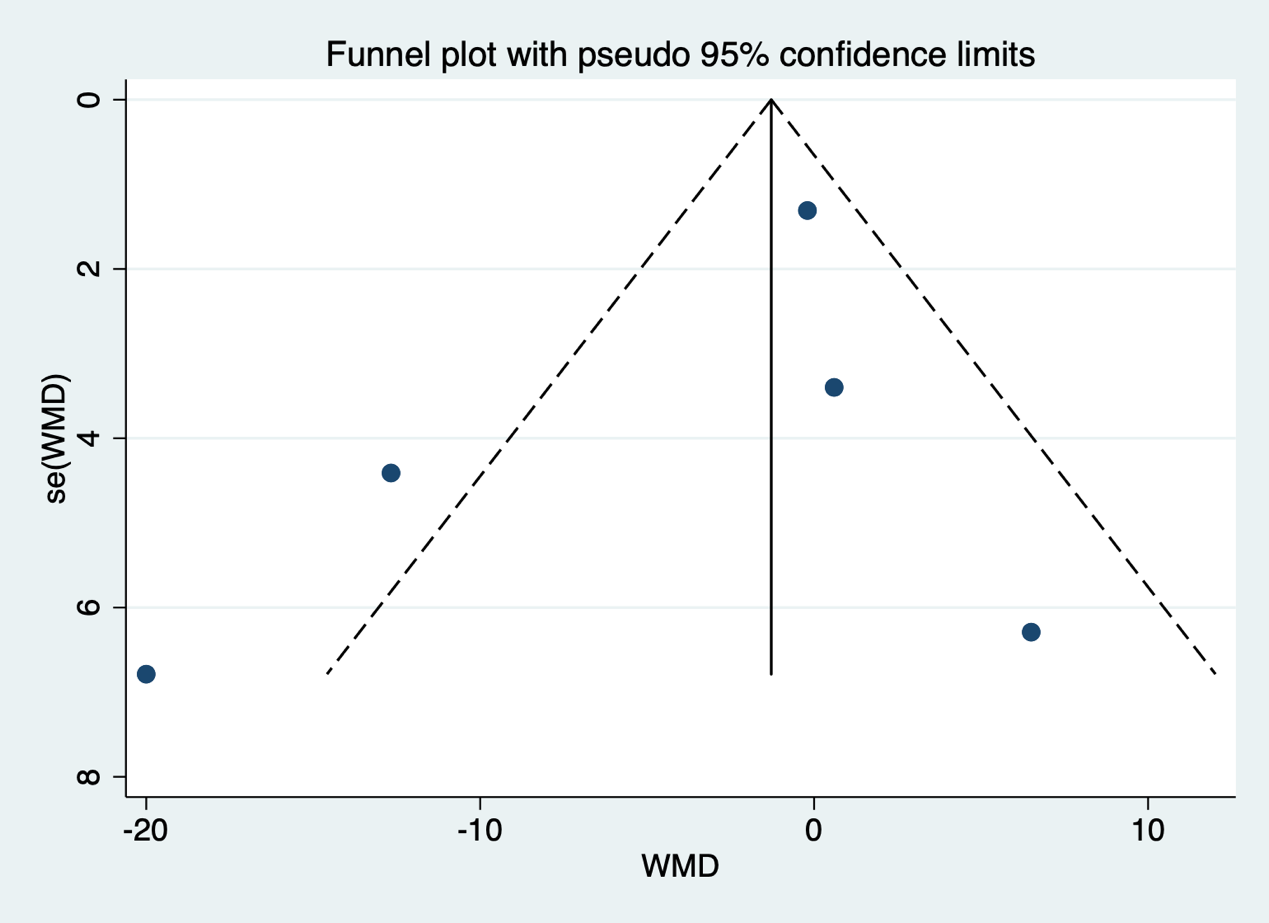


Figure S12 Funnel plot of the meta-analysis of SBP

Figure S13 Funnel plot of the meta-analysis of DBP


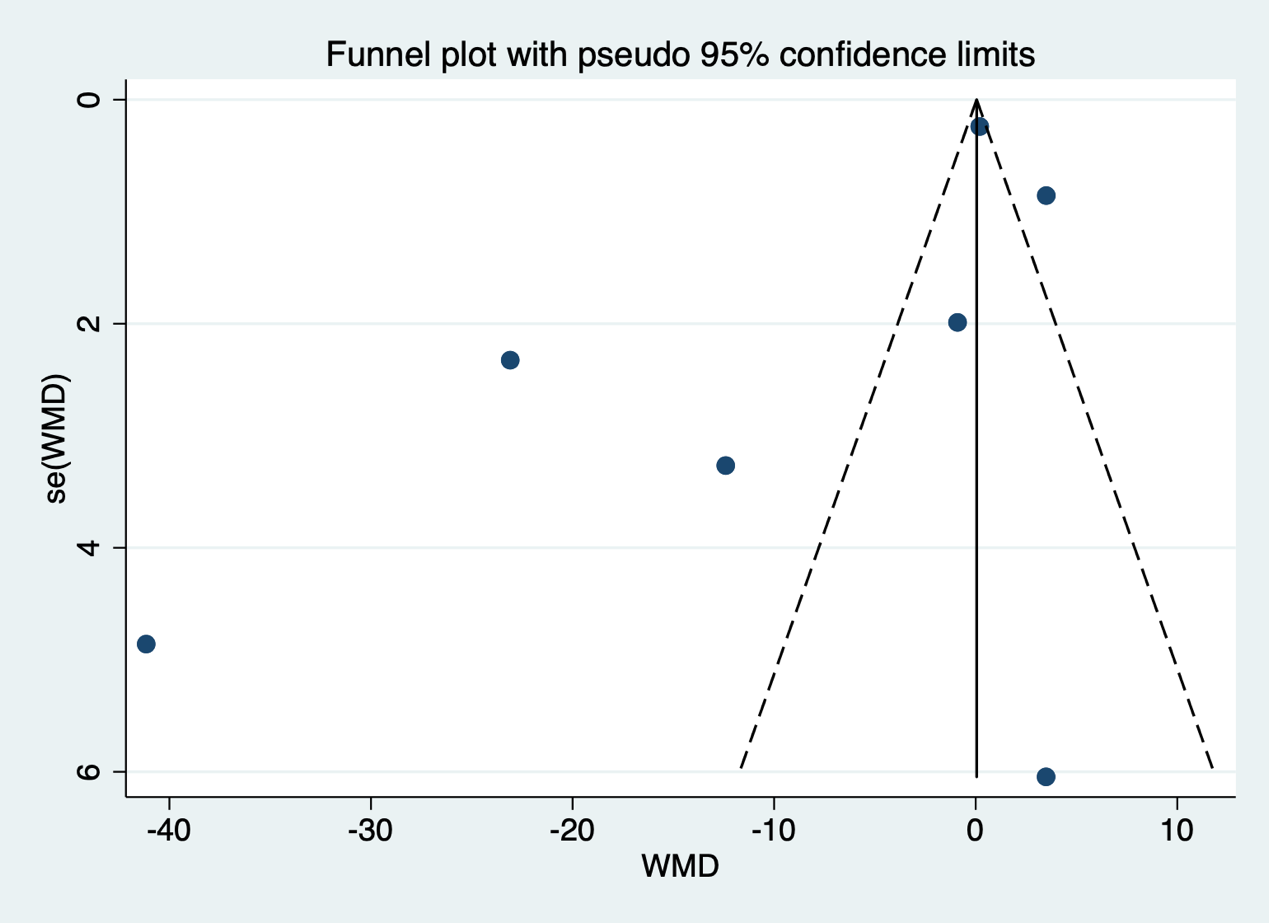


Figure S14 Funnel plot of the meta-analysis of LDL


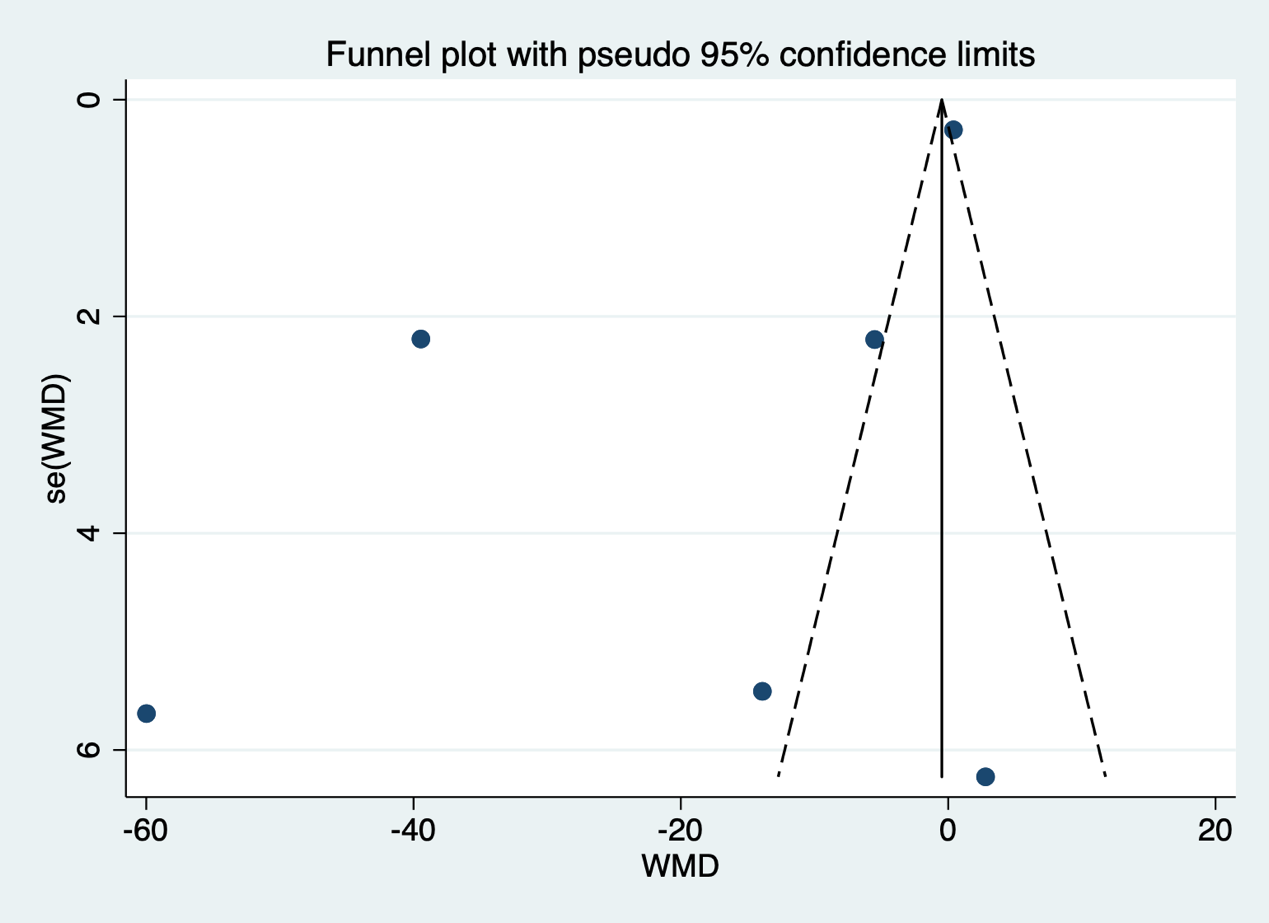


Figure S15 Funnel plot of the meta-analysis of TC


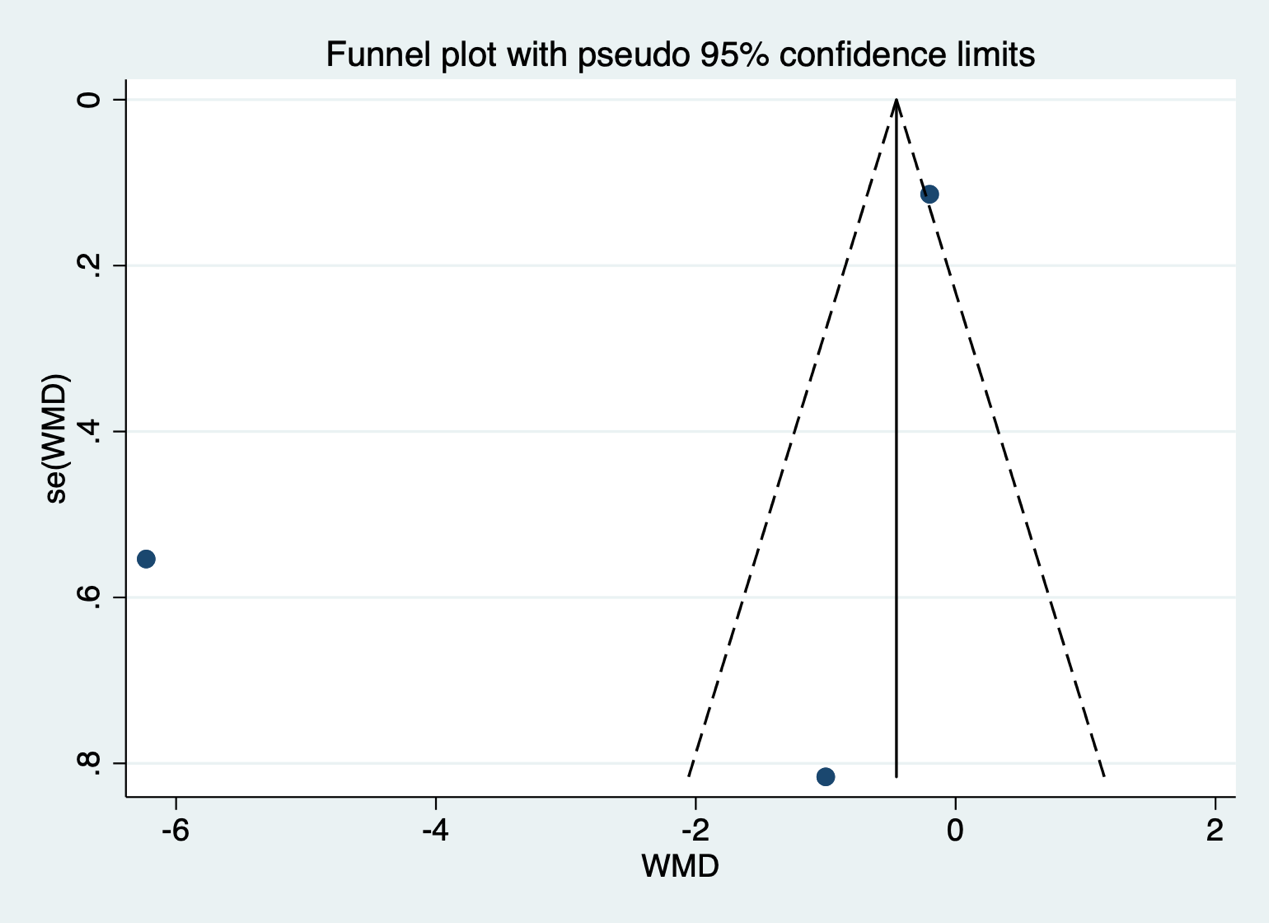


Figure S16 Funnel plot of the meta-analysis of IL-6


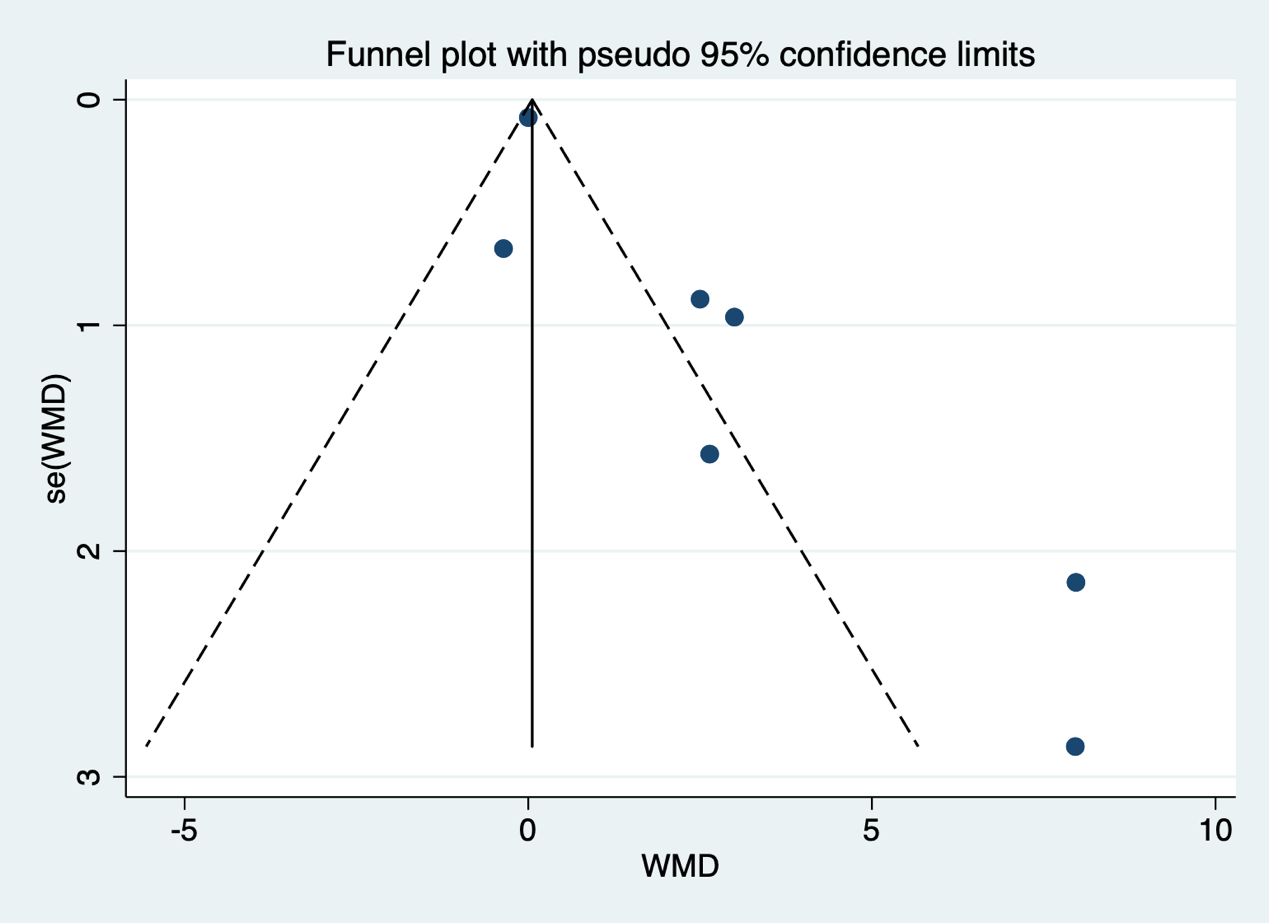


Figure S17 Funnel plot of the meta-analysis of HDL


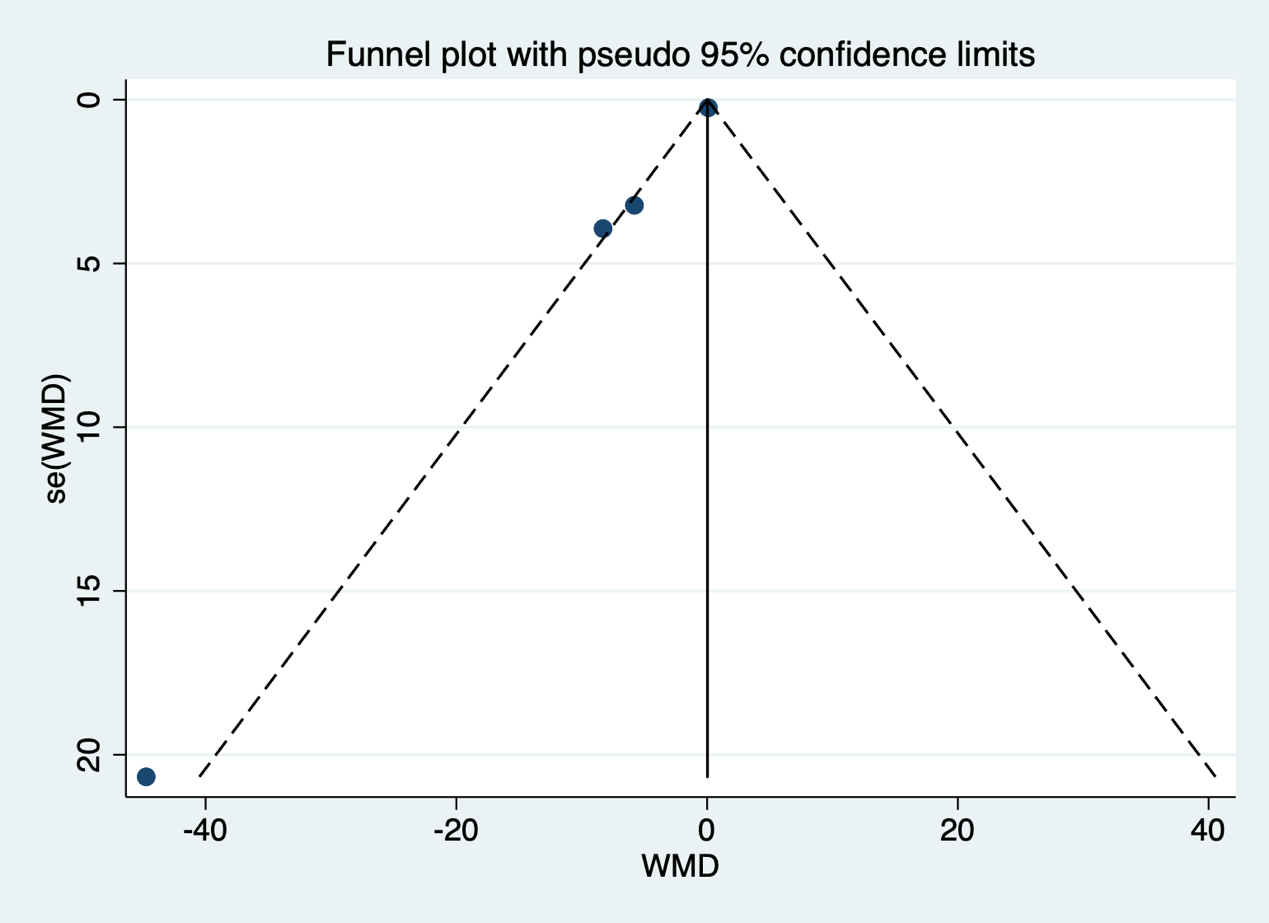


Figure S18 Funnel plot of the meta-analysis of TG
